# Universal Genetic Testing for Newly Diagnosed Invasive Breast Cancer

**DOI:** 10.1001/jamanetworkopen.2024.31427

**Published:** 2024-09-03

**Authors:** Zoulikha Rezoug, Stephanie P. Totten, David Szlachtycz, Adrienne Atayan, Kristen Mohler, Sophie Albert, Leila Feng, Brianna Lemieux Anglin, Zhen Shen, Daniel Jimenez, Nancy Hamel, Nicholas Meti, Khashayar Esfahani, Jean-François Boileau, Ipshita Prakash, Mark Basik, Sarkis Meterissian, Francine Tremblay, David Fleiszer, Dawn Anderson, George Chong, Stephanie M. Wong, William D. Foulkes

**Affiliations:** 1 Cancer Axis, Lady Davis Institute of the Jewish General Hospital, McGill University, Montréal, Québec, Canada; 2 Cancer Research Program, Research Institute of the McGill University Health Centre, McGill University, Montréal, Québec, Canada; 3 Department of Biomedical Sciences, College of Health Sciences, QU Health, Qatar University, Doha, Qatar; 4 Department of Human Genetics, McGill University, Montreal, Quebec, Canada; 5 Optilab-McGill University Health Centre, Montreal, Quebec, Canada; 6 Gerald Bronfman Department of Oncology, McGill University, Montreal, Quebec, Canada; 7 Department of Oncology, St Mary’s Hospital, McGill University, Montréal, Québec, Canada; 8 Department of Surgery, McGill University, Montreal, Quebec, Canada

## Abstract

**Importance:**

Between 5% and 10% of breast cancer cases are associated with an inherited germline pathogenic or likely pathogenic variant (GPV) in a breast cancer susceptibility gene (BCSG), which could alter local and systemic therapy recommendations. Traditional genetic testing criteria misses a proportion of these cases.

**Objective:**

To evaluate the prevalence and clinicopathological associations of GPVs in 2 groups of BCSGs among an ethnically diverse cohort of women with newly diagnosed breast cancer.

**Design, Setting, and Participants:**

This cross-sectional study, conducted at 3 Montreal hospitals between September 2019 and April 2022, offered universal genetic counseling and testing to all women with a first diagnosis of invasive breast cancer. Women were offered an obligatory primary panel of *BRCA1*, *BRCA2*, and *PALB2* (*B1B2P2*) and an optional secondary panel of 14 additional BCSGs. Eligible participants were women 18 years of age or older who received a diagnosis of a first primary invasive breast cancer not more than 6 months before the time of referral to the study. Data were analyzed from November 2023 to June 2024.

**Results:**

Of 1017 referred patients, 805 were eligible and offered genetic counseling and testing, and 729 of those 805 (90.6%) consented to be tested. The median age at breast cancer diagnosis was 53 years (range, 23-91 years), and 65.4% were White and of European ancestry. Fifty-four GPVs were identified in 53 patients (7.3%), including 39 patients (5.3%) with *B1B2P2* and 15 patients (2.1%) with 6 of the 14 secondary panel BCSGs (*ATM*, *BARD1*, *BRIP1*, *CHEK2*, *RAD51D*, and *STK11*). On multivariable analysis, clinical factors independently associated with *B1B2P2*-positive status included being younger than 40 years of age at diagnosis (odds ratio [OR], 6.83; 95% CI, 2.22-20.90), triple-negative breast cancer (OR, 3.19; 95% CI, 1.20-8.43), high grade disease (OR, 1.68; 95% CI, 1.05-2.70), and family history of ovarian cancer (OR, 9.75; 95% CI, 2.65-35.85). Of 39 *B1B2P2*-positive patients, 13 (33.3%) were eligible for poly(adenosine diphosphate–ribose) polymerase (PARP) inhibitors.

**Conclusions and Relevance:**

In this cross-sectional universal genetic testing study of women with newly diagnosed invasive breast cancer, the prevalence of GPVs was 7.3%, with 5.3% of patients testing positive for *B1B2P2*. Among *B1B2P2*-women women, one-third were eligible for PARP inhibitors.

## Key Points

**Question:**

What is the prevalence of germline pathogenic variants in breast cancer susceptibility genes among women with newly diagnosed invasive breast cancer?

**Findings:**

In this cross-sectional study of 729 female patients with a first diagnosis of breast cancer who participated in a universal genetic testing program, 5.3% had germline pathogenic variants in *BRCA1*/2 or *PALB2*, and 1.8% were considered eligible for poly(adenosine diphosphate–ribose) polymerase inhibitors based on their genetic testing result.

**Meaning:**

Findings suggest that universal genetic testing identifies actionable germline pathogenic variants in more than 1 in 20 patients with newly diagnosed breast cancer and is associated with systemic therapy recommendations in one-third of these cases.

## Teaser

This cross-sectional study evaluates the prevalence and clinicopathological associations of germline pathogenic or likely pathogenic variants in 2 groups of breast cancer susceptibility genes among a racially and ethnically diverse cohort of women with newly diagnosed breast cancer.

## Introduction

The use of multigene cancer susceptibility panels has revealed that 5% to 10% of women with breast cancer tested for these genes are found to carry a germline pathogenic or likely pathogenic variant (GPV).^1,2^ As the cost of genetic testing has decreased,^3^ the pressure to relax genetic testing criteria has increased.^4,5^ This pressure has only become more insistent now that effective first-line systemic therapies for *BRCA1* and *BRCA2* heterozygotes with early-stage or metastatic disease have become available.^6^

As existing cancer genetics services cannot provide counseling and genetic testing in a timely fashion in response to this demand, it has been argued that treating physicians should offer genetic testing to a broader group of women diagnosed with breast cancer, ideally at diagnosis.^7,8^ This process, referred to as *mainstreaming*,^5^ generally involves limited or no pretest genetic counseling, with positive results being handled by treating physicians, and subsequent referral to genetics health care professionals who then counsel affected women and offer appropriate follow-up with cascade testing of family members.^9^ Universal genetic testing (ie, without the need for meeting prespecified personal or family history criteria) was first implemented for patients with nonmucinous ovarian carcinoma^10,11,12,13,14,15^ and is now being extended to patients with invasive breast cancer.^16,17^ The results of these studies are beginning to be published^18,19^ and suggest broad acceptability of this approach by patients and health care professionals.

To address feasibility and evaluate optimal selection criteria, we performed a cross-sectional, multicentered universal genetic testing study of female patients with a first primary invasive breast cancer. The primary objective of this study was to determine the prevalence of GPVs in *BRCA1*, *BRCA2*, and *PALB2* (*B1B2P2*) as well as in other breast cancer susceptibility genes (BCSGs) within a racially and ethnically diverse cohort of women with newly diagnosed breast cancer.

## Methods

### Study Design, Setting, and Patient Selection

All women aged 18 years or older with a first diagnosis of pathologically confirmed, stage I to stage IV breast cancer between September 2019 and April 2022 at 1 of 3 McGill University–affiliated institutions (McGill University Cedars Cancer Centre; Jewish General Hospital Segal Cancer Centre; St Mary’s Hospital Cancer Centre) in Montreal, Canada, were eligible for inclusion. Patients with a personal history of breast cancer diagnosed greater than 6 months prior to study referral, in situ malignant tumor, or prior genetic testing for hereditary breast or ovarian cancer or women diagnosed at outside institutions without central pathology review were deemed ineligible (Figure). Eligible patients were referred by treating oncologists and contacted to offer an appointment for genetic counseling, including a 3-generation pedigree with self-reported racial and ethnic origins. Following pretest counseling, written informed consent was obtained from those who elected to proceed, and a blood sample was collected. Testing had 2 components; the first was an obligatory primary panel for *B1B2P2*, the 3 genes most likely to affect management. All patients were then offered optional testing in a secondary panel of 14 genes—*ATM*, *BARD1*, *BRIP1*, *CDH1*, *CHEK2*, *MLH1*, *MSH2*, *MSH6*, *PMS2*, *PTEN*, *RAD51C*, *RAD51D*, *STK11*, and *TP53*.

**Figure.**
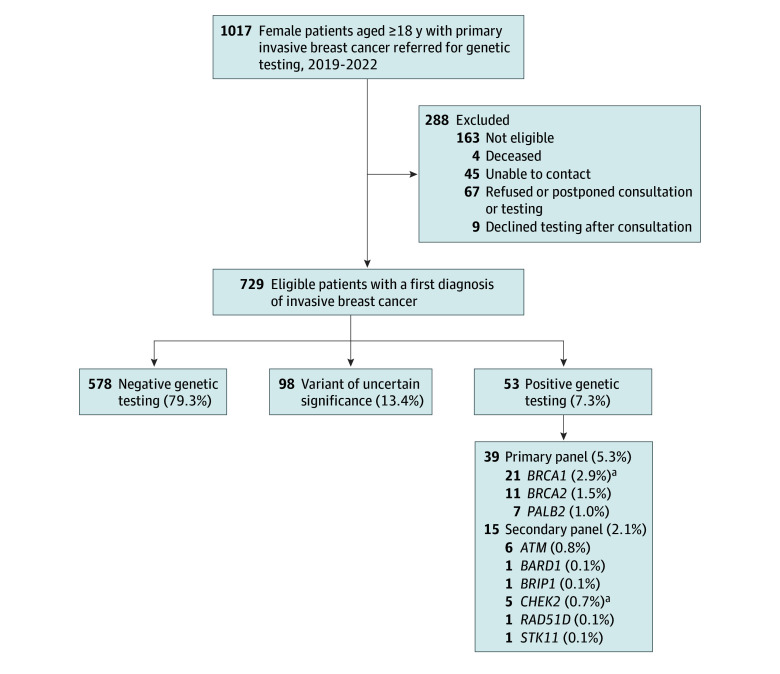
Cohort Selection and Genetic Testing Results Of 729 women tested, 53 (7.3%) were identified with 54 germline pathogenic variants. ^a^A single patient had 2 pathogenic or likely pathogenic variants in *BRCA1* and *CHEK2*.

Phase 1, which ran from September 2019 to April 2021, did not have an upper age limit for eligibility. On interim review of our data at a COVID-19–related recruitment pause, we amended the protocol such that affected women older than 70 years at diagnosis, except those with triple-negative breast cancer (TNBC), were deemed ineligible. Thus, phase 2 was implemented, starting October 2021, until study completion in April 2022. The study was approved by the research ethics boards at McGill University Health Centre and integrated university health and social services centres (ie, CIUSSS West-Central hospitals). The study adhered to the Strengthening the Reporting of Observational Studies in Epidemiology (STROBE) reporting guideline for cross-sectional studies.

### Molecular Analysis

DNA from the patient’s blood sample was enriched for targeted regions using a hybrid capture-based protocol and sequenced on an Illumina MiSeq platform. The following transcripts were used in this analysis: ATM (NM_000051.4), BARD1 (NM_000465.4), BRCA1 (NM_007294.4), BRCA2 (NM_000059.4), BRIP1 (NM_032043.3), CDH1 (NM_004360.5), CHEK2 (NM_007194.4), MLH1 (NM_000249.4), MSH2 (NM_000251.3), MSH6 (NM_000179.3), PALB2 (NM_024675.4), PMS2 (NM_000535.7), PTEN (NM_000314.8), RAD51C (NM_058216.3), RAD51D (NM_002878.4), STK11 (NM_000455.4), and TP53 (NM_000546.6). Variant calling was performed using NextGENe, version 2.4.2.3 and Geneticist Assistant, version 1.8.1 (SoftGenetics) proprietary bioinformatics pipeline. The fastq files from the Illumina MiSeq were aligned to hg19, and variant calling was performed on the resulting BAM files. For copy number variants, we used VarSeq (Golden Helix).

### Variant Classification

Variants were annotated according to the American College of Medical Genetics 5-tiered categorization as pathogenic, likely pathogenic, variant of uncertain significance, likely benign, or benign using Clinvar; VarSome, release 11.9; Franklin by Genoox; and the Human Gene Mutation Database (2019). Variants that were annotated as benign or likely benign were removed. Follow-up consultations with a cancer geneticist (W.D.F.) and a certified genetic counselor (Z.R. or A.A.) was offered to persons identified to have GPVs. W.D.F. reviewed all genetic testing results and decided whether to report the variant of uncertain significance or not. Each variant of uncertain significance was assessed on its own merit, with attention paid to the likely clinical utility of the variant in the context of the personal and family history of cancer. Founder variants associated with a less than 2-fold increased odds of breast cancer—that is, *CHEK2* c.470C>T (p.I157T) and c.1283C>T (p.S428F)—were categorized as variants of uncertain significance.

### Statistical Analysis

Statistical analysis was performed from November 2023 to June 2024. All patient and genetic-sequencing information was collected and managed using REDCap electronic data capture tools and LabKey software, version 21.11.11.^20^ Patient characteristics and genetics data were collected throughout phases 1 and 2, with additional variables on treatment and outcomes collected between May 2022 to October 2023. The χ^2^ test, the Fisher exact test, and the Wilcoxon rank sum test were used to compare patients who underwent the full 17-gene panel vs those testing for *B1B2P2* only, as well as to perform univariate analyses to evaluate the association between clinical characteristics and a GPV in the primary and secondary panels. Univariate logistic regression and multivariable logistic regression were then performed to determine factors independently associated with a GPV, with incorporation of all significant variables on univariate analysis into the adjusted multivariable model unless there was significant colinearity between variables (ie, stage and tumor size or nodal status) or the presence of a composite variable (ie, testing criteria that included age, high-risk ancestry, family history, and biologic subtype, which were already present within the model). If no factors were found to be significant on univariate analyses, univariate logistic regression was performed to calculate odds ratios (ORs) and corresponding 95% CIs. Analyses were carried out from November 2023 to June 2024 using SAS software, version 9.4 (SAS Institute Inc). The χ^2^ test was used to calculate *P* values, and all *P* values were 2-sided, with *P* < .05 used to indicate statistical significance.

## Results

Following the initial referral of 1017 female patients with breast cancer, 805 eligible patients were offered genetic counseling, and 729 (90.6% of those eligible) underwent pretest counseling followed by testing (Figure). Of 729 patients, 659 (90.4%) opted to receive the primary and secondary panel, while 70 (9.6%) elected to receive primary panel testing for *B1B2P2* only. There were no significant differences between those who accepted and those who refused the secondary panel with respect to median age, race, high-risk (Ashkenazi Jewish) ancestry, or family history of breast cancer, ovarian cancer, or other cancers (all *P* > .05).

### Cohort Characteristics

Within the testing cohort of 729 patients, the median age at diagnosis was 53 years (range, 23-91 years); 477 patients (65.4%) were White or of European ethnicity, with 54 (7.4%) of Ashkenazi Jewish ancestry and 167 (22.9%) of French-Canadian ancestry (Table 1). Of these 729 patients, 49 (6.7%) had a first- or second-degree family history of ovarian cancer with or without breast cancer, and 297 (40.7%) had a first- or second-degree family history of breast cancer without ovarian cancer. Most women in the cohort presented with estrogen receptor (ER)–positive, ERBB2 (formerly HER2 or HER2/neu)–negative breast cancer (487 of 729 [66.8%]), while 15.4% of the cohort (112 of 729) had TNBC. Overall, 214 patients (29.4%) met traditional risk-based criteria for genetic testing based on age at diagnosis, family history, and high-risk ancestry (eTable 1 in Supplement 1).

**Table 1.** Cohort Characteristics

Characteristic	Patients, No. (%) (N = 729)
Age at diagnosis, median (range), y	53 (23-91)
Race and ethnicity	
White or European	477 (65.4)
Black, African, or Caribbean	32 (4.4)
Asian or Southeast Asian	76 (10.4)
Hispanic or South or Central American	22 (3.0)
Middle Eastern or North African	70 (9.6)
Indigenous or First Nations	3 (0.4)
Mixed or unknown	49 (6.7)
Ancestry	
Ashkenazi Jewish	54 (7.4)
French Canadian	167 (22.9)
Other (non–Ashkenazi Jewish or non–French Canadian) or unknown	508 (69.7)
Family history	
No known family history of cancer	164 (22.5)
Ovarian cancer (with or without breast cancer)	49 (6.7)
Breast cancer (without ovarian cancer)	297 (40.7)
Any nonbreast or nonovarian cancer	219 (30.4)
Laterality	
Unilateral	702 (96.3)
Bilateral	27 (3.7)
Histology	
Invasive ductal carcinoma	584 (80.1)
Invasive lobular carcinoma	73 (10.0)
Mixed invasive ductal or lobular carcinoma	32 (4.4)
Other or unknown histology	40 (5.5)
Grade	
I	114 (15.6)
II	386 (53.0)
III	229 (31.4)
Biologic subtype	
ER positive, ERBB2 negative	487 (66.8)
ERBB2 positive	130 (17.8)
TNBC	112 (15.4)
Clinical tumor size	
cT1	389 (53.4)
cT2	264 (36.2)
cT3-T4	67 (9.2)
Unknown	9 (1.2)
Clinical nodal status	
cN0	541 (74.2)
cN1	159 (21.8)
cN2-N3	23 (3.2)
Unknown	6 (0.8)
Anatomic stage at presentation	
I	345 (47.3)
II	299 (41.0)
III	49 (6.7)
IV	28 (4.0)
Unknown	8 (1.1)

Abbreviations: ER, estrogen receptor; TNBC, triple-negative breast cancer.

### Genetic Testing Results

Fifty-four GPVs in the BCSGs were identified in 53 of 729 patients (7.3%), including 39 (5.3%) in *B1B2P2* and 15 (2.1%) in 6 of the remaining 14 genes (*ATM*, *BARD1*, *BRIP1*, *CHEK2*, *RAD51D*, and *STK11*) (eTable 2 and eTable 3A and B in Supplement 1). One patient had a GPV in both *BRCA1* and *CHEK2*. The distributions of variants identified for *B1B2P2*, *CHEK2*, and *ATM* are shown in eFigure 1 in Supplement 1. Of 729 patients, 98 (13.4%) had a variant of uncertain significance (eTable 4 in Supplement 1). Ethnicity and the distribution of GPVs and variants of uncertain significance by parental origin are shown in eTable 5 and eFigure 2 in Supplement 1.

Of the 659 patients who elected to receive testing in all 17 genes, 35 (5.3%) had a GPV in *B1B2P2* (including 1 patient with a GPV in both *BRCA1* and *CHEK2*), while 14 (2.1%) had a GPV in 1 of *ATM*, *BARD1*, *BRIP1*, *CHEK2*, *RAD51D*, or *STK11* (Table 2). The remaining 610 patients (92.6%) had no GPV identified on the full panel (hereafter referred to as *noncarriers*). Notably, testing for *ATM*, *BRCA1*, *BRCA2*, *CHEK2*, and *PALB2* identified 93% of all GPVs found in the 17-gene panel. Patients with a GPV in *B1B2P2* on the primary panel were younger (median age, 42 years [range, 29-79 years]) than noncarriers (median age, 53 years [range, 23-91 years]) and women with GPVs in the secondary panel (median age, 58 years [range, 36-69]; *P* = .03). There was no difference in race and ethnicity, ancestry, laterality, histology, or clinical nodal status between women with GPV in the primary or secondary panel and those who tested negative (Table 2). Women with a GPV in *B1B2P2* were more likely to have a family history of ovarian cancer (7 of 35 [20.0%]) compared with noncarriers (34 of 610 [5.6%]) or those with a GPV in secondary panel genes (2 of 14 [14.3%]; *P* = .02). They were also more likely to demonstrate high-grade disease (23 of 35 [65.7%]) compared with noncarriers (176 of 610 [28.9%]) and those with a GPV in secondary panel genes (4 of 14 [28.6%]; *P* < .001), as well as TNBC (18 of 35 [51.4%] vs 80 of 610 noncarriers [13.1%] vs 1 of 14 patients with GPVs on secondary panel [7.1%]; *P* < .001). In contrast, women with GPVs in secondary panel genes had a higher likelihood of hormone-sensitive breast cancer (10 of 14 ER-positive, ERBB2-negative women [71.4%]) similar to that of noncarriers (418 of 610 ER-positive, ERBB2-negative noncarriers [68.5%]). Overall, 12 of 35 *B1B2P2* GPV carriers (34.3%) and 12 of 14 secondary panel GPV carriers (85.7%) would not have met traditional risk-based criteria for genetic testing compared with 440 of 610 noncarriers (72.1%) (*P* < .001).

**Table 2.** Clinical Characteristics by Genetics Result of All Women Who Underwent 17-Gene Panel Testing

Characteristic	Patients, No. (%) (n = 659)			*P* value
	GPV on primary panel (n = 35)^a^	GPV on secondary panel (n = 14)^b^	No GPV detected (n = 610)	
Age at diagnosis, median (range), y	42 (29-79)	58 (36-69)	53 (23-91)	.03
Race and ethnicity				
White or European	19 (54.3)	11 (78.6)	402 (66.0)	.47
Black, African, or Caribbean	1 (3.5)	0	28 (4.6)	
Asian or Southeast Asian	6 (17.1)	1 (7.1)	59 (9.7)	
Hispanic or South or Central American	3 (8.6)	0	19 (3.1)	
Middle Eastern or North African	5 (14.3)	0	58 (9.5)	
Indigenous or First Nations	0	0	2 (0.3)	
Mixed or unknown	1 (2.9)	2 (14.3)	42 (6.9)	
Ancestry				
Ashkenazi Jewish	1 (2.9)	0	46 (7.4)	.69
French Canadian	9 (25.7)	4 (28.6)	140 (23.1)	
Other (non-Ashkenazi Jewish or non–French Canadian) or unknown	25 (71.4)	10 (71.4)	424 (69.5)	
Family history				
No known family history of cancer	6 (17.1)	2 (14.3)	135 (22.1)	.02
Ovarian cancer (with or without breast cancer)	7 (20.0)	2 (14.3)	34 (5.6)	
Breast cancer	16 (45.7)	5 (35.7)	249 (40.8)	
Any nonbreast or nonovarian cancer	6 (17.1)	5 (35.7)	192 (31.5)	
Laterality				
Unilateral	35 (100.0)	13 (92.9)	586 (96.1)	.40
Synchronous bilateral	0	1 (7.1)	24 (3.9)	
Histology				
Invasive ductal carcinoma	33 (94.3)	10 (71.4)	482 (79.0)	.29
Invasive lobular carcinoma	0	2 (14.3)	65 (10.7)	
Mixed invasive ductal or lobular carcinoma	2 (5.7)	1 (7.1)	28 (4.6)	
Other or unknown histology	0	1 (7.1)	35 (5.7)	
Grade				
I	0	2 (14.3)	104 (17.1)	<.001
II	12 (34.3)	8 (57.1)	330 (54.1)	
III	23 (65.7)	4 (28.6)	176 (28.9)	
Biologic subtype				
ER positive, ERBB2 negative	14 (40.0)	10 (71.4)	418 (68.5)	<.001
ERBB2 positive	3 (8.6)	3 (21.4)	112 (18.4)	
TNBC	18 (51.4)	1 (7.1)	80 (13.1)	
Clinical tumor size				
cT1	11 (31.4)	9 (69.2)	343 (56.7)	.04
cT2	20 (57.1)	3 (23.1)	211 (34.9)	
cT3-T4	4 (11.4)	1 (7.7)	51 (8.4)	
Clinical nodal status^c^				
cN0	22 (62.9)	10 (71.4)	459 (75.6)	.40
cN1	12 (34.3)	3 (21.4)	128 (21.1)	
cN2-N3	1 (2.9)	1 (7.1)	20 (3.3)	
Anatomic stage at presentation^c^				
I	8 (22.9)	9 (69.2)	305 (50.5)	.02
II	24 (68.6)	3 (23.1)	236 (39.1)	
III	2 (5.7)	1 (7.7)	42 (7.0)	
IV	1 (2.9)	0	21 (3.5)	
Met traditional testing criteria				
No	12 (34.3)	12 (85.7)	440 (72.1)	<.001
Yes	23 (65.7)	2 (14.3)	170 (27.9)	

Abbreviations: ER, estrogen receptor; GPV, germline pathogenic or likely pathogenic variant; TNBC, triple-negative breast cancer.

^a^ The 3-gene panel included *BRCA1/2* and *PALB2* genes; 1 patient with germline pathogenic variants in both *BRCA1* and *CHEK2* was included in this group.

^b^ The 14-gene panel included *ATM*, *BARD1*, *BRIP1*, *CDH1*, *CHEK2*, *MLH1*, *MSH2*, *MSH6*, *PMS2*, *PTEN*, *RAD51C*, *RAD51D*, *STK11*, and *TP53*.

^c^ Patients with unknown data were excluded from χ^2^ analysis.

### Clinical Factors Associated With GPVs in *BRCA1/2* and *PALB2* and Secondary Panel Genes

On univariate analysis, young age, family history of ovarian cancer, histology, histologic grade, biologic subtype, tumor size, and stage were significantly associated with a GPV on the primary panel of *B1B2P2* (Table 3). Despite the expected higher frequency of *BRCA1/2* GPVs in the Ashkenazi Jewish population, we did not see this in this study as only 3 of 54 Ashkenazi Jewish patients were positive for GPVs in these 2 genes. Younger than 40 years of age, 16 of 88 patients tested (18.2%) positive for *B1B2P2* compared with 10 of 196 patients (5.1%) aged 40 to 49 years and 6 of 227 patients (2.6%) aged 50 to 59 years (*P* < .001). Of 112 patients with TNBC, 22 (19.6%) tested positive for *B1B2P2* compared with only 14 of 487 ER-positive, ERBB2-negative patients (2.9%) and 3 of 130 ERBB2-positive patients (2.3%) (*P* < .001). Among patients with TNBC diagnosed at younger than 40 years of age, 41.7% (10 of 24) had a GPV in *B1B2P2*, decreasing to 29.4% (5 of 17) for those 40 to 49 years of age, 13.8% (4 of 29) for those 50 to 59 years, 7.1% (2 of 28) for those 60 to 69 years, and 7.1% (1 of 14) for those older than 70 years. On multivariable logistic regression, the factors that remained independently associated with *B1B2P2* included age, family history of ovarian cancer, histologic grade, and biologic subtype. Relative to patients aged 50 to 59 years, those diagnosed with breast cancer at younger than 40 years had a 6.8-fold increased likelihood of carrying a GPV in *B1B2P2* (OR, 6.83; 95% CI, 2.22-20.90), and compared with patients with no family history of cancer, those with a family history of ovarian cancer had a nearly 10-fold increased odds (OR, 9.75; 95% CI, 2.65-35.85). In terms of breast cancer clinical features, high-grade histology increased the odds of a GPV in *B1B2P2* by 68% (OR, 1.68; 95% CI, 1.05-2.70), and compared with ER-positive, ERBB2-negative breast cancers, TNBC was associated with a 3-fold increase in the odds of a GPV in *B1B2P2* (OR, 3.19; 95% CI, 1.20-8.43).

**Table 3.** P/LP Variants in *BRCA1/2* or *PALB2* Among Women With Newly Diagnosed Invasive Breast Cancer

Characteristic	Cohort (N = 729)	Patients with P/LP variant on primary panel (n = 39)		Adjusted OR (95% CI) for P/LP variant in *BRCA1/2* or *PALB2*^a^
	No. (%)	No. (%)	*P* value	
Age group, y				
<40	88 (12.1)	16 (18.2)	<.001	6.83 (2.22 20.90)^b^
40-49	196 (26.9)	10 (5.1)		2.40 (0.76-7.53)
50-59	227 (31.1)	6 (2.6)		1.00 [Reference]
60-69	154 (21.1)	4 (2.6)		1.00 (0.26-3.91)
≥70	63 (8.6)	3 (4.8)^c^		1.78 (0.38-8.24)
Race and ethnicity				
White or European	477 (65.4)	23 (4.8)	.36	NA
Black, African, or Caribbean	32 (4.4)	1 (3.1)		
Asian or Southeast Asian	76 (10.4)	6 (7.9)		
Hispanic or South or Central American	22 (3.0)	3 (13.6)		
Middle Eastern or North African	70 (9.6)	5 (7.1)		
Indigenous or First Nations	3 (0.4)	0		
Other or unknown	49 (6.7)	1 (2.1)		
Ancestry				
Ashkenazi Jewish	54 (7.4)	3 (5.6)	.91	NA
French Canadian	167 (22.9)	10 (6.0)		
Other or unknown	508 (69.7)	26 (5.1)		
Family history				
No known family history of cancer	164 (22.5)	6 (3.7)	<.001	1.00 [Reference]
Ovarian cancer (with or without breast cancer)	49 (6.7)	8 (16.7)		9.75 (2.65-35.85)^b^
Breast cancer	297 (40.7)	19 (6.4)		2.47 (0.87-7.03)
Any nonbreast or nonovarian cancer	219 (30.0)	6 (2.7)		0.90 (0.26-3.14)
Laterality				
Unilateral	702 (96.3)	39 (5.6)	.39	NA
Synchronous bilateral	27 (3.7)	0		
Histology				
Invasive ductal carcinoma	584 (80.1)	37 (6.4)	.02	1.00 [Reference]
Mixed or invasive lobular carcinoma or other	145 (19.9)	2 (1.4)		0.33 (0.07-1.48)
Histologic grade				
I-II	500 (68.6)	12 (2.4)	<.001	1.00 [Reference]
III	229 (31.4)	27 (11.8)		1.68 (1.05-2.70)^b^
Biologic subtype				
ER positive, ERBB2 negative	487 (66.8)	14 (2.9)	<.001	1.00 [Reference]
ERBB2 positive	130 (17.8)	3 (2.3)		0.35 (0.09-1.41)
TNBC	112 (15.4)	22 (19.6)		3.19 (1.20-8.43)^b^
Clinical tumor size				
cT1	389 (54.0)	13 (3.3)	.03	NA
cT2	265 (36.8)	20 (7.6)		
cT3-cT4	67 (9.3)	6 (9.0)		
Clinical nodal status				
cN0	542 (74.9)	26 (4.8)	.34	NA
cN1	159 (22.0)	12 (7.6)		
cN2-cN3	23 (3.2)	1 (4.4)		
Stage				
I	345 (47.9)	10 (2.9)	.02	1.00 [Reference]
II	299 (41.0)	25 (8.4)		1.73 (0.73-4.07)
III	49 (6.7)	2 (4.1)		0.77 (0.13-4.39)
IV	28 (3.8)	2 (7.1)		1.10 (0.19-6.26)
Met traditional testing criteria				
No	515 (70.6)	12 (2.4)	<.001	NA
Yes	214 (29.4)	27 (12.7)		

Abbreviations: ER, estrogen receptor; NA, not applicable; OR, odds ratio; P/LP, pathogenic or likely pathogenic; TNBC, triple-negative breast cancer.

^a^ Multivariable logistic regression was performed with incorporation of all significant variables on univariate analysis into the adjusted model, with adjusted ORs and 95% CIs reported.

^b^ Statistically significant at *P* < .05.

^c^ Enriched for patients with TNBC in phase 2 of the study (eTable 8 in Supplement 1); 3.6% of all subtypes were included in phase 1 of the study.

For the 659 patients who underwent secondary panel testing, no specific clinical factors were significantly associated with GPVs identified in *ATM*, *BARD1*, *BRIP1*, *CHEK2*, *CDH1*, *MSH2*, *MSH6*, *MLH1*, *PTEN*, *RAD51C*, *RAD51D*, or *TP53* (all *P* > .05) (eTable 6 in Supplement 1). However, the study was not specifically powered to detect associations between demographic or clinical features and GPVs in these genes. Only 34% of patients positive for *B1B2P2* but 86% of patients positive for the other BCSGs would not have been eligible for genetic testing according to traditional risk-based criteria used in the regular medical genetics service (eTable 1 in Supplement 1), so it is clear that the high-risk criteria are, as expected, biased toward identifying *B1B2P2* heterozygotes and were not designed to (and in fact are less capable of) identifying persons carrying lower-risk GPVs (eTable 6 in Supplement 1).^1,21^

### Eligibility for Poly(Adenosine Diphosphate–Ribose) Polymerase Inhibitors

Prior to genetic testing, 101 of 729 patients (13.9%) with early-stage and metastatic breast cancer would have been candidates for poly(adenosine diphosphate–ribose) polymerase (PARP) inhibitors based on biologic subtype, clinical stage, pathologic stage, and/or response to neoadjuvant chemotherapy (eTable 7 in Supplement 1). Overall, most PARP inhibitor candidates had TNBC (64 of 729 [8.8% of total cohort]), predominantly those with residual disease after neoadjuvant chemotherapy (54 of 729 [7.4% of total cohort]), while the remaining 37 of 729 PARP inhibitor candidates (5.1% of total cohort) had ER-positive, ERBB2-negative breast cancer.

Of the 64 PARP inhibitor candidates with TNBC, 12 (18.8%) tested positive for a *BRCA1/2* GPV, whereas of 37 PARP inhibitor candidates with ER-positive, ERBB2-negative breast cancer, 1 (2.7%) tested positive for a *BRCA1* GPV, and none were positive for *BRCA2*. Thus, in the overall cohort of 729 patients, 13 (1.8% of total cohort) had a confirmed GPV in *BRCA1/2* and were eligible for PARP inhibitors. Of these women, 12 (92.3%) met traditional institutional criteria for genetic testing, while 1 (7.7%), a 67-year-old woman with high-grade TNBC, fell outside traditional testing criteria.

## Discussion

Over the past decade, restrictive genetic testing criteria have gradually loosened as improved technology^3^ and legal rulings^22^ have driven testing costs down. Moreover, the development of PARP inhibitor therapies specifically targeting tumors with homologous recombination repair deficiency^6,23^ has motivated a wholesale review of the process by which women affected by breast cancer receive genetic testing.^24^ Thus, new models of genetic testing are emerging in breast cancer genetics,^25^ and mainstreaming is becoming increasingly popular as the demand for testing cannot be met by existing genetics services.^8^ The question, however, of whether all women with invasive breast cancer should be offered publicly funded early genetic testing for BCSGs^4^ is unresolved, and there is a paucity of data on which to make decisions in this sphere.

Here, we show in this large cross-sectional study that from a cohort of 729 women with incident breast cancer, 53 (7.3%) carried a GPV in a BCSG, including 5.3% with a GPV in *B1B2P2*. These percentages are in line with the prevalence of GPVs in women reported in previous breast cancer studies in which more relaxed testing criteria were used (Table 4).^1,2,18,19,26,27,28^ As expected, on multivariable analysis, clinical factors significantly associated with GPVs in the 3 major BCSGs (*BRCA1*, *BRCA2*, and *PALB2*) included being younger than 40 years at diagnosis, women with TNBC, and those with a family history of ovarian cancer. If we wished to identify 95% of all GPVs in these 3 genes, then testing all women younger than 50 years, all patients with TNBC, and all those with a family history of breast or ovarian cancer would achieve this goal, with 95% of patients with *B1B2P2* identified and 543 tests performed. Simplifying criteria by testing all women with TNBC or breast cancer at younger than 65 years of age would result in identification of 92.3% of patients with *B1B2P2* and 634 tests performed, whereas a single age cutoff of breast cancer diagnosed at or younger than 70 years of age would yield identical identification rates but with the highest number of tests (n = 673) required.

**Table 4.** Comparison of Universal Genetic Testing Studies

Characteristic	Tung et al,^2^ 2016	Beitsch et al,^1^ 2019		Yadav et al,^26^ 2020	Shelton et al,^19^ 2024	De Silva et al,^18^ 2023	This study
No. of patients	488	959		3907	192	474	729
% of Patients invited who consented	69.8	Not reported		84.9	91.4	89.7^a^	90.6
Study design	Retrospective single-institution study of prospectively collected specimens from patients with breast cancer in Massachusetts	Prospective multicentered registry; 20 community and academic centers in the US		Retrospective single-institution study from prospectively collected specimens within a registry or biobank (Mayo Clinic Breast Cancer Study) conducted in Minnesota^b^	Prospective single-institution study conducted in a rural setting in North Carolina	Prospective single-institution study (Mutational Assessment of Newly Diagnosed Breast Cancer Using Germline and Tumour Genomics) conducted in Melbourne, Australia	Prospective, multicentered study (Genetic Rapid Easy Access Testing) conducted in Montreal, Canada
Inclusion criteria	All women with a first diagnosis of stage I-III breast cancer who consented for DNA banking	All women aged 18-90 y, currently or previously treated for breast cancer		All women with a first diagnosis of invasive breast cancer or ductal carcinoma in situ	All patients diagnosed with unilateral, stage 0-III breast cancer, including male patients (n = 6 [5.8%])	All women with newly diagnosed, stage I-III breast cancer, as well as high-grade ductal carcinoma in situ or pleomorphic lobular carcinoma in situ	All women aged ≥18 y with a first diagnosis of invasive breast cancer
Exclusion criteria	Prior breast cancer	Prior genetic testing		Prior diagnosis of breast cancer; lobular carcinoma in situ; samples available but failed sequencing; insufficient family or personal history to allow for evaluation based on NCCN criteria	NA	Metastatic breast cancer; prior genetic testing; prior identification of hereditary breast or ovarian cancer pathogenic variant	Prior diagnosis of invasive breast cancer; ductal or lobular carcinoma in situ; prior genetic testing; pathologically unconfirmed disease
No. of genes tested	25^c^	80^d^		9^e^	Variable (median, 47)^f^	19^g^	17^h^
Frequency of P/LP variants, % of total cohort	10.7	8.65		6.2	13	6.5	7.3
Genes with pathogenic or likely pathogenic variants identified	*ATM*, *BRCA1*, *BRCA2*, *BRIP1*, *CHEK2*, *NBN*, *MSH6*, *PALB2*, *PMS2*, *PTEN*	*ATM*, *BARD1*, *BLM*, *BRCA1*, *BRCA2*, *CHEK2*, *DIS3L2*, *FH*, *MITF*, *MSH6*, *MUTYH*, *NBN*, *NF1*, *NTHL1*, *PALB2*, *PTEN*, *RAD50*, *RAD51C*, *RAD51D*, *RB1*, *RECQL4*, *RET*, *TP53*, *VHL*, *WRN*		*ATM*, *BRCA1*, *BRCA2*, *CDH1*, *CHEK2*, *NF1*, *PALB2*, *PTEN*, *TP53*	*ATM*, *BARD1*, *BRCA1*, *BRCA2*, *CHEK2*, *FANCC*, *MSH3*, *MUTYH*, *NTHL1*, *NF1*, *PALB2*, *PMS2*, *RAD50*, *RAD51C*, *RECQL4*	*ATM*, *BARD1*, *BRCA1*, *BRCA2*, *CHEK2*, *MSH6*, *RAD51C*, *PALB2*, *PMS2*	*ATM*, *BARD1*, *BRCA1*, *BRCA2*, *BRIP1*, *CHEK2*, *PALB2*, *RAD51D*, *STK11*
% of Patients aged >65 y with P/LP variant	NA	8.8		1.7	NA	NA	6.8
% of P/LP variant carriers aged >65 y	9.6	28.8		7.9	NA	22.5	13.8
Frequency of P/LP variants in *BRCA1/*2 and *PALB2*, % of total cohort	6.4	2.4		3.1	4.2	3.2	5.3
Race and ethnicity, No. (%)	Non-Hispanic (not Ashkenazi Jewish) White, 397 (81.4); African American, 12 (2.5); Asian, 10 (2.0); Hispanic,17 (3.5); other, 14 (2.9)	White, 772 (80.0); Black or African American, 63 (6.6); Asian, 65 (6.8); multiracial, 27 (2.8); Native American or Alaska Native, 6 (0.6); Native Hawaiian or Other Pacific Islander, 11 (1.2); unknown, 15 (1.6)		NA	White, 184 (95.8); Black or African American, 4 (2.1); Asian, 3 (1.5); Hispanic or Latino, 2 (1.0)	NA	White, 477 (65.4); Black, African, or Caribbean, 32 (4.4); Asian or South East Asian, 76 (10.4); Hispanic or South or Central American, 22 (3.0); Middle Eastern or North African, 70 (9.6); Indigenous or First Nations, 3 (0.4); other or unknown, 49 (6.7)
Ancestry, No. (%)	Ashkenazi Jewish, 38 (7.8)	NA		NA	NA	NA	Ashkenazi Jewish, 54 (7.4); French Canadian, 167 (22.9); other or unknown, 508 (69.7)
% of P/LP variants in patients who did not meet testing criteria per guidelines	NA	7.9 (NCCN)		3.5 (NCCN)	11.4 (NCCN)	NA	6.0 (Institutional guidelines; eTable 1 in Supplement 1)
% of Patients found to have a P/LP variant who did not meet testing criteria	NA	44 (NCCN)		29.7 (NCCN)	40 (NCCN)	58 (CanRisk or Manchester Score, >10%; Australian guidelines)	49.0

Abbreviations: NA, not available; P/LP, pathogenic or likely pathogenic.

^a^ During initial pilot study (National Comprehensive Cancer Network [NCCN]).

^b^ The Mayo Clinic Breast Cancer Study was 1 of the studies contributing to the Cancer Risk Estimates Related to Susceptibility consortium.^27^

^c^ *APC*, *ATM*, *BARD1*, *BMPR1A*, *BRCA1*, *BRCA2*, *BRIP1*, *CDH1*, *CDK4*, *CDKN2A*, *CHEK2*, *EPCAM*, *MLH1*, *MSH2*, *MSH6*, *MUTYH*, *NBN*, *PALB2*, *PMS2*, *PTEN*, *RAD51C*, *RAD51D*, *SMAD4*, *STK11*, *TP53*.

^d^ *ALK*, *APC*, *ATM*, *AXIN2*, *BAP1*, *BARD1*, *BLM*, *BMPR1A*, *BRCA1*, *BRCA2*, *BRIP1*, *CASR*, *CDC73*, *CDH1*, *CDK4*, *CDKN1B*, *CDKN1C*, *CDKN2A*, *CEBPA*, *CHEK2*, *DICER1*, *DIS3L2*, *EGFR*, *EPCAM*, *FH*, *FLCN*, *GATA2*, *GPC3*, *GREM1*, *HOXB13*, *HRAS*, *KIT*, *MAX*, *MEN1*, *MET*, *MITF*, *MLH1*, *MSH2*, *MSH6*, *MUTYH*, *NBN*, *NF1*, *NF2*, *PALB2*, *PDGFRA*, *PHOX2B*, *PMS2*, *POLD1*, *POLE*, *POT1*, *PRKAR1A*, *PTCH1*, *PTEN*, *RAD50*, *RAD51C*, *RAD51D*, *RB1*, *RECQL4*, *RET*, *RUNX1*, *SDHA*, *SDHAF2*, *SDHB*, *SDHC*, *SDHD*, *SMAD4*, *SMARCA4*, *SMARCB1*, *SMARCE1*, *STK11*, *SUFU*, *TERC*, *TERT*, *TMEM127*, *TP53*, *TSC1*, *TSC2*, *VHL*, *WRN*, *WT1*.

^e^ *ATM*, *BRCA1*, *BRCA2*, *CDH1*, *CHEK2*, *NF1*, *PALB2*, *PTEN*, *TP53*.

^f^ Complete list is not reported.

^g^ *ATM*, *BARD1*, *BRCA1*, *BRCA2*, *BRIP1*, *CDH1*, *CHEK2*, *MLH1*, *MSH2*, *MSH6*, *NTHL1*, *PALB2*, *PMS2*, *PTEN*, *RAD51B*, *RAD51C*, *RAD51D*, *STK11*, *TP53*.

^h^ *ATM*, *BARD1*, *BRCA1*, *BRCA2*, *BRIP1*, *CDH1*, *CHEK2*, *MLH1*, *MSH2*, *MSH6*, *PALB2*, *PTEN*, *RAD51C*, *RAD51D*, *STK11*, *TP53.*

Recent consensus panel guidelines for germline testing for patients with breast cancer suggest that women who fall outside age-based criteria should be offered *BRCA1/2 t*esting if they are candidates for PARP inhibitor therapy for early-stage or metastatic disease.^29^ Candidacy is based on eligibility criteria derived from clinical trials for *BRCA*-associated metastatic breast cancer,^30,31^ as well as eligibility criteria from OlympiA for early-stage disease.^6^ In our study, 18.8% of PARP inhibitor candidates with TNBC and 2.7% of PARP inhibitor candidates with ER-positive, ERBB2-negative breast cancer tested positive for a GPV in *BRCA1/2*. Alternatively, one-third of *BRCA1/2* carriers were eligible for PARP inhibitors, of whom 4 (30.8%) were older than 50 years and 1 (7.7%) was older than 65 years with TNBC. Thus, testing criteria that include all patients with TNBC as well as these age-specific thresholds are likely to capture most PARP inhibitor–eligible patients with *BRCA1/2,* with a small volume of additional testing required for PARP inhibitor candidates who do not meet these criteria.

We offered both an immediately actionable panel of 3 established BCSGs (*B1B1P2*) as well as a secondary panel of 14 BCSGs. More than 90% of participants opted for the larger panel, but, in fact, testing for only 5 moderate- to high-risk genes (*ATM*, *BRCA1*, *BRCA2*, *CHEK2*, and *PALB2*) identified 93% of all the GPVs found using our 17-gene panel. While many commercial laboratories offer large multigene cancer predisposition panels, these and other recent results^18,28^ suggest that settling for a panel with well-known moderate- to high-risk genes will detect the substantial majority of all relevant GPVs, and exceptionally large panels will increase the prevalence of variants of uncertain significance without identifying large numbers of GPVs relevant to the current diagnosis.

In this study, we did not identify any clincopathologic factors significantly associated with GPVs in the secondary gene panel (eTable 6 in Supplement 1). Moreover, given the lack of actionability associated with GPVs in many of the secondary genes, it is questionable whether testing for these BCSGs is warranted in a mainstreaming setting, where the prior probability of identifying a GPV is low. Prior large studies of patients undergoing multigene panel testing have, however, identified modest associations with early-onset breast cancer and GPVs in *ATM* and *CHEK2*,^32^ and it should be emphasized that our study was not specifically powered to detect demographic or clinical factors associated with a GPV in these and other secondary panel genes.

Differences in breast cancer subtypes in distinct population groups have been reported.^33^ Here, the prevalence of GPVs was not different between racial ethnic groups (Table 3). In contrast, certain unstudied Global South populations are more likely to report variants of uncertain significance than are Global North populations, and in some Global South regions, the proportion of variants of uncertain significance substantially outnumbers the proportion of GPVs identified (eTable 7 and eFigure 2A and B in Supplement 1), emphasizing the need for sequencing data from these populations.^34,35,36^ As a point of reference, much knowledge on breast cancer predisposition was initially gathered from Ashkenazi Jewish and European-origin populations. From previous data, one would expect at least 10% of affected Ashkenazi Jewish women to carry 1 of 3 common *BRCA1/2* founder GPVs in this population.^37^ Here, we found that only 3.7% of Ashkenazi Jewish women with breast cancer were carriers of *BRCA1* c.68_69del (1 woman carried a nonfounder *BRCA1* variant). This reduced percentage could be due to the long-standing high level of hereditary risk awareness that exists in the Montreal Ashkenazi Jewish community.^38^ Many predisposed families have already benefitted from cascade testing and risk-reducing strategies.

### Strengths and Limitations

The study has strengths and limitations. Offering to test all women led to a more complete picture of the distribution of GPVs across ages and races and ethnicities. Referral biases were largely eliminated. Nevertheless, introducing a new breast cancer predisposition genetic counseling and testing model, at the start of the COVID-19 pandemic, combined with the existing constraints of a public health system, led to challenges in completing aspects of the project in a timely manner. It was due to these significant hurdles, 18 months into the study, that we paused recruitment for 6 months to assess these factors. Recruitment was adjusted to focus on patients with a new diagnosis who were younger than 70 years, unless their diagnosis was a TNBC, in which case, age was not a consideration. Although there were no significant clinical differences noted between the 525 and 204 patients tested within phase 1 and phase 2 of the study, apart from age distribution (eTable 8 in Supplement 1), this resulted in patients 70 years of age or older comprising only 3.4% of the cohort tested in phase 2 compared with 10.7% of those tested in phase 1. Moreover, these restrictions have resulted in an overestimation of the GPV prevalence among women older than 70 years, as this group was enriched for TNBC in phase 2 of the study (eTable 9 in Supplement 1). Notably, in phase 1 of the study, 56 patients older than 70 years underwent genetic testing with a 3.6% prevalence of *B1B2P2* compared with a 14.3% prevalence of *B1B2P2* among the 7 women aged 70 years or older with TNBC tested in phase 2.

## Conclusions

In this cross-sectional universal genetic testing study of women with newly diagnosed invasive breast cancer, 7.3% had a GPV in a BCSG, with 5.3% of patients testing positive for *B1B2P2*. Of those testing positive for *BRCA1*/2 or *PALB2*, one-third were eligible for PARP inhibitors. The results of this study and related studies^18,28^ have informed our clinical practice, and we now offer mainstream, oncology-led genetic testing to all women diagnosed with incident invasive breast cancer younger than 50 years of age, those with TNBC and/or bilateral breast cancer, those potentially eligible for PARP inibitors, and, unrelated to this study, male patients with breast cancer. Affected women who do not meet these criteria are referred to the medical genetics service for appropriate evaluation. As genetic testing evolves, publicly funded genetic testing programs will need to be evaluated for benefit and cost-effectiveness in clinical situations.

## Notes

Supplement 1. **eTable 1.** Traditional Hereditary Breast and Ovarian Cancer Testing Criteria Used at McGill Hospitals **eTable 2.** Germline Pathogenic and Likely Pathogenic Variants (n=729) **eTable 3A.** Cases With a Pathogenic or Likely Pathogenic Variant **eTable 3B.** Patients With a Pathogenic/Likely Pathogenic Variants That Were Not Found To Be Disease Causing Due To a Low Allele Frequency in Blood DNA and Absence From the Breast Cancer Tumor **eTable 4.** Variants of Uncertain Significance (n=98) **eTable 5.** Distribution of Germline Pathogenic/Likely Pathogenic Variants and Variants of Uncertain Significance by Parental Origin **eTable 6.** Predictors of Pathogenic or Likely Pathogenic Variants in Secondary 14-Gene Panel Among Women With Newly Diagnosed Invasive Breast Cancer (n=659) **eTable 7.** PARP Inhibitor Eligibility **eTable 8.** Clinical Characteristics of Patients Stratified by Phase I and Phase II of the Study (n=729) **eTable 9.** Biologic Subtype by Age Group (n=729) **eFigure 1A.** Lollipop Plot for *BRCA1* Germline Variants **eFigure 1B.** Lollipop Plot for *BRCA2* Germline Variants **eFigure 1C.** Lollipop Plot for *PALB2* Germline Variants **eFigure 1D.** Lollipop Plot for *ATM* Germline Variants **eFigure 1E.** Lollipop Plot for *CHEK2* Germline Variants **eFigure 2.** World Map Showing Ethnicity of Patients With a Pathogenic Variant or Variant of Uncertain Significance, per Region
eReferences.

Supplement 2.
Data Sharing Statement
