# Supplementary material for: Universal Genetic Testing for Newly Diagnosed Invasive Breast Cancer
Source: JAMA Netw Open. 2024 Sep 3;7(9):e2431427. doi: 10.1001/jamanetworkopen.2024.31427 (PMC11372499; doi:10.1001/jamanetworkopen.2024.31427)
Supplement: Supplement 1. — eTable 1. Traditional Hereditary Breast and Ovarian Cancer Testing Criteria Used at McGill Hospitals eTable 2. Germline Pathogenic and Likely Pathogenic Variants (n=729) eTable 3A. Cases With a Pathogenic or Likely Pathogenic Variant eTable 3B. Patients With a Pathogenic/Likely Pathogenic Variants That Were Not Found To Be Disease Causing Due To a Low Allele Frequency in Blood DNA and Absence From the Breast Cancer Tumor eTable 4. Variants of Uncertain Significance (n=98) eTable 5. Distribution of Germline Pathogenic/Likely Pathogenic Variants and Variants of Uncertain Significance by Parental Origin eTable 6. Predictors of Pathogenic or Likely Pathogenic Variants in Secondary 14-Gene Panel Among Women With Newly Diagnosed Invasive Breast Cancer (n=659) eTable 7. PARP Inhibitor Eligibility eTable 8. Clinical Characteristics of Patients Stratified by Phase I and Phase II of the Study (n=729) eTable 9. Biologic Subtype by Age Group (n=729) eFigure 1A. Lollipop Plot for BRCA1 Germline Variants eFigure 1B. Lollipop Plot for BRCA2 Germline Variants eFigure 1C. Lollipop Plot for PALB2 Germline Variants eFigure 1D. Lollipop Plot for ATM Germline Variants eFigure 1E. Lollipop Plot for CHEK2 Germline Variants eFigure 2. World Map Showing Ethnicity of Patients With a Pathogenic Variant or Variant of Uncertain Significance, per Region eReferences. [file jamanetwopen-e2431427-s001.pdf]

Supplemental Online Content

Rezoug Z, Totten SP, Szlachtycz D, et al. Universal genetic testing for newly diagnosed invasive breast cancer. *JAMA Netw Open*. 2024;7(9):e2431427. doi:10.1001/jamanetworkopen.202431427

eTable 1. Traditional Hereditary Breast and Ovarian Cancer Testing Criteria Used at McGill Hospitals
eTable 2. Germline Pathogenic and Likely Pathogenic Variants (n=729)
eTable 3A. Cases With a Pathogenic or Likely Pathogenic Variant
eTable 3B. Patients With a Pathogenic/Likely Pathogenic Variants That Were Not Found To Be Disease Causing Due To a Low Allele Frequency in Blood DNA and Absence From the Breast Cancer Tumor
eTable 4. Variants of Uncertain Significance (n=98)
eTable 5. Distribution of Germline Pathogenic/Likely Pathogenic Variants and Variants of Uncertain Significance by Parental Origin
eTable 6. Predictors of Pathogenic or Likely Pathogenic Variants in Secondary 14-Gene Panel Among Women With Newly Diagnosed Invasive Breast Cancer (n=659)
eTable 7. PARP Inhibitor Eligibility
eTable 8. Clinical Characteristics of Patients Stratified by Phase I and Phase II of the Study (n=729)
eTable 9. Biologic Subtype by Age Group (n=729)
eFigure 1A. Lollipop Plot for BRCA1 Germline Variants
eFigure 1B. Lollipop Plot for BRCA2 Germline Variants
eFigure 1C. Lollipop Plot for PALB2 Germline Variants
eFigure 1D. Lollipop Plot for ATM Germline Variants
eFigure 1E. Lollipop Plot for CHEK2 Germline Variants
eFigure 2. World Map Showing Ethnicity of Patients With a Pathogenic Variant or Variant of Uncertain Significance, per Region
eReferences.

This supplemental material has been provided by the authors to give readers additional information about their work.

© 2024 Rezoug Z et al. *JAMA Network Open.*

eTable 1. Traditional hereditary breast and ovarian cancer testing criteria used at McGill Hospitals

**Individual with…**	
	Breast cancer diagnosed under age 35
	Triple negative breast cancer diagnosed under age 50
	Two primary breast cancers, at least one diagnosed under age 50
	Ovarian cancer diagnosed at any age
	Male breast cancer diagnosed under age 60
	Patient of Ashkenazi Jewish ancestry with ovarian cancer at any age or breast cancer diagnosed under age 65
**Family* with…**	
	BRCA1 or *BRCA2* mutation identified
	Breast cancer in 3 close relatives with at least one diagnosed under age 50
	Breast cancer in 2 close relatives under age 50
	1 first-degree relative with both breast and ovarian cancer at any age
	Breast cancer under age 60 and close relative with ovarian cancer at any age
	Male breast cancer and close relative with breast cancer under age 60 or ovarian cancer at any age
	2 close relatives with ovarian cancer at any age
	Ashkenazi Jewish ancestry with family history of ovarian cancer at any age OR breast cancer under age 65

Breast cancer refers to all invasive cancers, all high grade ductal carcinoma in situ (DCIS) or DCIS under age 40 (excludes lobular carcinoma in situ/LCIS);
Ovarian cancer refers to all invasive non-mucinous epithelial cancer, primary peritoneal and fallopian tube cancer (excludes borderline/low malignant potential [LMP] ovarian tumours;
*Family referral criteria requires affected relatives be on the same side of the family;
†Close relatives include all first, second and third-degree relatives.

© 2024 Rezoug Z et al. *JAMA Network Open.*

eTable 2. Germline Pathogenic and Likely Pathogenic Variants (n=729)

Gene	No. patients	% of all tested*	% of P/LP variants
**Primary panel**			
BRCA1†	21	2.88	38.9
BRCA2	11	1.50	20.4
PALB2	7	0.96	13.0
**Secondary panel***			
ATM	6	0.90	11.1
CHEK2†	5	0.76	9.25
BARD1	1	0.15	1.85
RAD51D	1	0.15	1.85
STK11	1	0.15	1.85
RAD51C	0	-	-
CDH1	0	-	-
TP53	0	-	-
PTEN	0	-	-
BRIP1	1	0.15	1.85
MLH1	0	-	-
MSH2	0	-	-
MSH6	0	-	-
PMS2	0	-	-

P/LP: Pathogenic/Likely pathogenic; *Of the total cohort, 659 patients consented to undergo testing in secondary panel genes; †One patient had both *BRCA1* and *CHEK2* pathogenic variant

© 2024 Rezoug Z et al. *JAMA Network Open.*

eTable 3A. Cases with a Pathogenic or Likely Pathogenic Variant

Sample(s)	*Age at Diagnosis*	*Population Group*		*Gene*	*Genomic Variant*	*mRNA reference sequence*	*cDNA Variant*	*Protein Reference Sequence*	*Amino Acid Variant*	*Founder P/LP Variant*	*Founder population [Reference]*	*ClinVar.ID*	*ClinGen. ID*	*rs*
		**Maternal*†**	**Paternal*†**											
REF00424	39	French Canadian	Ashkenazi Jewish	*BRCA1*	17:41276044(ACTCT>ACT)	NM_007294.4	c.68_69del	*NP_009225.1*	p.Glu23fs	Y	AJ [1]	17662	CA003783	rs80357914
REF00830	71	Ashkenazi Jewish	Ashkenazi Jewish	*BRCA1*	17:41276044(ACTCT>ACT)	NM_007294.4	c.68_69del	NP_009225.1	p.Glu23fs	Y	AJ [1]	17662	CA003783	rs80357914
REF01028	28	Ukrainian	Ukrainian	*BRCA1*	17:41258504(A>C)	NM_007294.4	c.181T>G	NP_009225.1	p.Cys61Gly	Y	U [2]	17661	CA001182	rs28897672
REF00288	38	French Canadian	French Canadian	*BRCA1*	17:41256945(G>A)	NM_007294.4	c.241C>T	NP_009225.1	p.Gln81Ter	Y	FC [3]	54565	CA001613	rs80357350
REF00662	55	Lebanese	Lebanese	*BRCA1*	17:41246869(C>A)	NM_007294.4	c.679G>T	NP_009225.1	p.Glu227Ter	N		252435	CA10585944	rs879255319
REF00214	34	French Canadian	English Canadian	*BRCA1*	17:41245721(ATTCTTT>ATT)	NM_007294.4	c.1823_1826del	NP_009225.1	p.Lys608fs	N		54361	CA001184	rs80357585
REF00468**	31	French Canadian	English Canadian	*BRCA1*	17:41245422(A>AT)	NM_007294.4	c.2125_2126insA	NP_009225.1	p.Phe709fs	Y	FC [4]	54469	CA001418	rs80357871
REF00466	42	Armenian	Armenian	*BRCA1*	17:41245135(ACT>A)	NM_007294.4	c.2411_2412del	NP_009225.1	p.Gln804fs	N		37466	CA001605	rs80357664
REF00813	57	French Canadian	French Canadian	*BRCA1*	17:41244710(GATAC>GAG)	NM_007294.4	c.2834_2836delinsC	NP_009225.1	p.Ser945fs	Y	FC [5]	54694	CA001852	rs386134270
REF00659	40	Iranian	Iranian	*BRCA1*	17:41244404(ACC>AC)	NM_007294.4	c.3143del	NP_009225.1	p.Gly1048fs	N		266338	CA10589781	rs886040100
REF00836	30	Romanian	Romanian, Hungarian	*BRCA1*	17:41243941(G>A)	NM_007294.4	c.3607C>T	NP_009225.1	p.Arg1203Ter	P	G [6]	17671	CA002305	rs62625308
REF00620	32	French Canadian	French Canadian	*BRCA1*	17:41243729(CT>C)	NM_007294.4	c.3959del	NP_009225.1	p.Ser1320fs	N		266421	CA10589710	rs886040179
REF00467	43	Filipino	Filipino	*BRCA1*	11:43076504(TTT>TT)	NM_007294.4	c.4467del	NP_009225.1	p.Glu1490fs	N		1687131		
REF00800	33	Indian	Indian	*BRCA1*	17:41226515(G>T)	NM_007294.4	c.4508C>A	NP_009225.1	p.Ser1503Ter	Y	IS [7]	55219	CA002889	rs80357437
REF00809	38	Columbian	Columbian	*BRCA1*	17:41226420(C>A)	NM_007294.4	c.4603G>T	NP_009225.1	p.Glu1535Ter	N		55236	CA002924	rs80357366
REF00008	45	Greek	Greek	*BRCA1*	17:41209134(C>T)	NM_007294.4	c.5212G>A	NP_009225.1	p.Gly1738Arg	Y	G [6]	55461	CA003363	rs80356937
REF00210	36	Moroccan	Moroccan	*BRCA1*	17:41209095(G>A)	NM_007294.4	c.5251C>T	NP_009225.1	p.Arg1751Ter	N		55480	CA003389	rs80357123
REF01039	42	Brazilian	Brazilian	*BRCA1*	17:41209079(TGGG>TGGGG)	NM_007294.4	c.5266dup	NP_009225.1	p.Gln1756fs	Y	Br [8]	17677	CA003404	rs80357906

© 2024 Rezoug Z et al. *JAMA Network Open.*

REF00235	51	Slovenian	Slovenian	*BRCA1*	17:41209079(TGGG>TGGGG)	NM_007294.4	c.5266dup	NP_009225.1	p.Gln1756fs	Y	S [9]	17677	CA003404	rs80357906
REF00794	45	Hongkonger	Hongkonger	*BRCA1*	17:41201187(A>G)	NM_007294.4	c.5357T>C	NP_009225.1	p.Leu1786Pro	N		91649	CA003521	rs398122697
REF00441	41	Italian	Italian	*BRCA1*	17:41199696(G>A)	NM_007294.4	c.5431C>T	NP_009225.1	p.Gln1811Ter	N		55577	CA003593	rs397509283
REF01032	44	English Canadian	Ashkenazi Jewish	*BRCA1*	17:41197784(G>A)	NM_007294.4	c.5503C>T	NP_009225.1	p.Arg1835Ter	N		55601	CA003674	rs41293465
REF00712	71	English Canadian	Dutch	*BRCA2*	13:32906804(C>CTTAG)	NM_000059.4	c.1189_1190insTTAG	NP_000050.3	p.Gln397fs	N		51080	CA011099	rs397515635
REF00166	62	Grenadian	Grenadian	*BRCA2*	13:32907375(CAAATAA>CAA)	NM_000059.4	c.1763_1766del	NP_000050.3	p.Asn588fs	N		51187	CA013129	rs80359303
REF00333	38	French Canadian	French Canadian	*BRCA2*	13:32911073(CAAAAAAA>CAAAAAAAA)	NM_000059.4	c.2588dup	NP_000050.3	p.Asn863fs	Y	FC [5]	37793	CA015689	rs80359335
REF00068	79	Indian	Indian	*BRCA2*	13:32912337(CTGT>CT)	NM_000059.4	c.3847_3848del	NP_000050.3	p.Val1283fs	N		37859	CA018922	rs80359405
REF00528	36	Filipino	Filipino	*BRCA2*	13:32912345(GAAAAAAA>GAAAAAA)	NM_000059.4	c.3860del	NP_000050.3	p.Asn1287fs	N		51545	CA018995	rs80359406
REF00844	55	Vincentian	Vincentian	*BRCA2*	13:32944620(TTAT>TC)	NM_000059.4	c.8414_8416delinsC	NP_000050.3	p.Leu2805fs	N		38159	CA025629	rs397507402
REF00968	32	French Canadian	French Canadian	*BRCA2*	13:32945137(AAGAGAG>AAGAG)	NM_000059.4	c.8537_8538del	NP_000050.3	p.Glu2846fs	Y	FC [5]	9328	CA025697	rs80359714
REF00080	47	French Canadian, Belgian	French Canadian, German	*BRCA2*	13:32945137(AAGAGAG>AAGAG)	NM_000059.4	c.8537_8538del	NP_000050.3	p.Glu2846fs	Y	FC [5]	9328	CA025697	rs80359714
REF00151	35	French Canadian	French Canadian	*BRCA2*	13:32953937(G>A)	NM_000059.4	c.9004G>A	NP_000050.3	p.Glu3002Lys	Y	FC [10]	38201	CA025920	rs80359152
REF00535	60	French Canadian	French Canadian	*BRCA2*	13:518_631(del)	NM_000059.4	Exon 7 deletion		Non coding	N				
REF00434	66	Romanian	Romanian	*PALB2*	16:23647356(ATCT>AT)	NM_024675.4	c.509_510del	NP_078951.2	p.Arg170fs	L	Po [11]^	126757	CA214889	rs515726123
REF00992	39	Lebanese	Turkish	*PALB2*	16:23647163(GT>GTT)	NM_024675.4	c.703dup	NP_078951.2	p.Thr235fs	N		924795		rs1967027806
REF01044	61	Italian	Italian	*PALB2*	16:23647108(TA>TAA)	NM_024675.4	c.758dup	NP_078951.2	p.Ser254fs	N		126769	CA294009	rs515726126
REF00927	37	Italian	Italian	*PALB2*	16:23647108(TA>TAA)	NM_024675.4	c.758dup	NP_078951.2	p.Ser254fs	N		126769	CA294009	rs515726126
REF00899	52	Venezuelan, Vietnamese	Venezuelan	*PALB2*	16:23641306(CAT>C)	NM_024675.4	c.2167_2168del	NP_078951.2	p.Met723fs	N		136132	CA164353	rs587776416
REF01042	51	Filipino	Filipino	*PALB2*	16:23637659(ACAACC>ACAACCAACC)	NM_024675.4	c.2642_2645dup	NP_078951.2	p.Cys882fs	N		182741	CA299663	rs730881868
REF00719	40	Algerian	Algerian	*PALB2*	16:1686_2514(del)	NM_024675.4	Exon 5 deletion			N				

© 2024 Rezoug Z et al. *JAMA Network Open.*

REF00261	58	English Canadian	English Canadian	*ATM*	11:108114696(C>G)	NM_000051.4	c.513C>G	NP_000042.3	p.Tyr171Ter	N		184787	CA190051	rs786201693
REF00679	61	Italian	Italian	*ATM*	11:108121752(CAGAGA>CAGA)	NM_000051.4	c.1564_1565del	NP_000042.3	p.Glu522fs	N		127340	CA273990	rs587779817
REF00818	56	French Canadian	Unknown	*ATM*	11:108121752(CAGAGA>CAGA)	NM_000051.4	c.1564_1565del	NP_000042.3	p.Glu522fs	N		127340	CA273990	rs587779817
REF00314	40	English Canadian	English Canadian	*ATM*	11:108155055(T>C)	NM_000051.4	c.3848T>C	NP_000042.3	p.Leu1283Pro	N		181994	CA298351	rs730881389
REF00906	36	Chinese	Chinese	*ATM*	11:108172385(C>T)	NM_000051.4	c.5188C>T	NP_000042.3	p.Arg1730Ter	N		189054	CA334791	rs764389018
REF00474	69	French Canadian	French Canadian	*ATM*	11:108236050(A>G)	NM_000051.4	c.8988-2A>G		Non coding	N		185326	CA191650	rs786202087
REF00264	56	Dominican	Dominican	*BARD1*	2:215617281(T>C)	NM_000465.4	c.1569-2A>G	NP_000456.2	Non Coding	N		845400		rs1693509336
REF00362	60	Bengali	Bengali	*BRIP1*	17:59770798(A>T)	NM_032043.3	c.2568T>A	NP_114432.2	p.Tyr856Ter	N		1459831		
REF01005	67	Slovenian, Yugoslavian	Slovenian, Hungarian	*CHEK2*	22:29121230(C>T)	NM_007194.4	c.444+1G>A		Non coding	L	Po [15]	128075	CA288309	rs121908698
REF00520	46	German, Spanish	German, other European	*CHEK2*	22:29099530(AG>A)	NM_007194.4	c.870del	NP_009125.1	p.Phe292fs	N		460863	CA658656848	rs1555916987
REF00296	53	English Canadian	English Canadian	*CHEK2*	22:29091856(AG>A)	NM_007194.4	c.1100del	NP_009125.1	p.Thr367fs	Y	Eu [12]	128042	CA288251	rs555607708
REF00468	31	French Canadian	English Canadian	*CHEK2*	22:29091856(AG>A)	NM_007194.4	c.1100del	NP_009125.1	p.Thr367fs	Y	FC [13]	128042	CA288251	rs555607708
REF00865	62	French Canadian	French Canadian	*CHEK2*	22:320_592 (dup)	NM_007194.4	Exon 3-4 duplication			N		583612		
REF01051	47	French Canadian	French Canadian	*RAD51D*	17:33430520(G>A)	NM_002878.4	c.620C>T	NP_002869.3	p.Ser207Leu	Y	FC [17]	142102	CA167404	rs370228071
REF00277	67	English Canadian	English Canadian	*STK11*	19:1219322(C>A)	NM_000455.4	c.375-1C>A		Non coding	N				

© 2024 Rezoug Z et al. *JAMA Network Open.*

eTable 3B. Patients with a pathogenic/likely pathogenic variants that were not found to be disease causing due to a low allele frequency in blood DNA and absence from the breast cancer tumor.

Sample(s)	***Age at Diagnosis***	***Population Group***		***Gene***	***Genomic Variant***	***mRNA reference sequence***	***cDNA Variant***	***Protein Reference Sequence***	***Amino Acid Variant***	***Founder P/LP Variant Y/N***	***ClinVar.ID***	***ClinGen. ID***	***rs***
		**Maternal**	**Paternal**										
REF00858	67	Greek	Greek	CHEK2	22:29121091(AAGAG>AAG)	NM_007194.3	c.464_465delCT	NP_009125.1	p.Ser155fs		2125994		
REF00951	53	Russian	Russian	ATM	11:(108203709_108204535)_(108204774_108205616)del	NM_000051.4	Exon 54 deletion	NP_000042.3	deletion Exon 54				

Germline pathogenic variants are ordered in terms of coding position; exon deletions and non-coding variants are listed at the end of each gene list.
*As reported by patients. †Ethnic groups referred to as ‘French Canadian’ and ‘English Canadian’ include Quebecers, Acadians and other historically French-speaking populations of French descent and the Canadian population of British Isles descent, respectively. Note**: REF00468 has two P/LP variants (one in *BRCA1*, and one in *CHEK2*);^Common haplotype not shown, however also seen in other Central and Eastern European countries [18] and to date, rarely in other populations, therefore is likely a founder. Founder P/LP variant: Y: Yes; N: No; L: Likely; P: Possible. AJ: Ashkenazi Jewish; B: Belarussian; Br Brazilian; Eu: European; FC: French Canadian; G: Greek; IS: Indian subcontinent; Po: Polish; S: Slovenian; U: Ukrainian

© 2024 Rezoug Z et al. *JAMA Network Open.*

eTable 4. Variants of uncertain significance (n=98)

Gene	Genomic Variant	Variant	Times Observed
ATM	11:108098614(A>G)	NM_000051.4(ATM):c.184A>G (p.Arg62Gly)	1
ATM	11:108115723(C>G)	NM_000051.4(ATM):c.871C>G (p.His291Asp)	1
ATM	11:108117735(T>C)	NM_000051.4(ATM):c.946T>C (p.Tyr316His)	1
ATM	11:108121588(C>G)	NM_000051.4(ATM):c.1396C>G (p.Gln466Glu)	1
ATM	11:108122682(A>G)	NM_000051.4(ATM):c.1726A>G (p.Ile576Val)	1
ATM	11:108124585(T>C)	NM_000051.4(ATM):c.1943T>C (p.Val648Ala)	1
ATM	11:108128232(A>G)	NM_000051.4(ATM):c.2275A>G (p.Ser759Gly)	1
ATM	11:108142025(T>C)	NM_000051.4(ATM):c.2969T>C (p.Ile990Thr)	1
ATM	11:108143552(G>A)	NM_000051.4(ATM):c.3257G>A (p.Arg1086His)	1
ATM	11:108155013(A>G)	NM_000051.4(ATM):c.3806A>G (p.Lys1269Arg)	1
ATM	11:108158382(C>T)	NM_000051.4(ATM):c.4049C>T (p.Thr1350Met)	1
ATM	11:108164118(C>T)	NM_000051.3(ATM):c.4690C>T (p.Pro1564Ser)	1
ATM	11:108165702(A>G)	NM_000051.4(ATM):c.4825A>G (p.Thr1609Ala)	1
ATM	11:108168084(C>A)	NM_000051.4(ATM):c.4980C>A (p.Asn1660Lys)	1
ATM	11:108175456(C>T)	NM_000051.4(ATM):c.5551C>T (p.Leu1851Phe)	1
ATM	11:108190757(A>G)	NM_000051.4(ATM):c.6424A>G (p.Thr2142Ala)	1
ATM	11:108192075(A>G)	NM_000051.4(ATM):c.6500A>G (p.Tyr2167Cys)	1
ATM	11:108196186(A>G)	NM_000051.4(ATM):c.6722A>G (p.Asn2241Ser)	1
ATM	11:108200949(T>C)	NM_000051.4(ATM):c.7316T>C (p.Val2439Ala)	1
ATM	11:108204685(T>C)	NM_000051.4(ATM):c.8000T>C (p.Met2667Thr)	1
ATM	11:108206576(G>A)	NM_000051.4(ATM):c.8156G>A (p.Arg2719His)	1
ATM	11:108216546(G>A)	NM_000051.4(ATM):c.8495G>A (p.Arg2832His)	1
ATM	11:108236192(A>G)	NM_000051.4(ATM):c.9128A>G (p.Lys3043Arg)	1
BARD1	2:215674297(C>T)	NM_000465.4(BARD1):c.-4G>A	1
BARD1	2:215674179(C>T)	NM_000465.4(BARD1):c.115G>A (p.Ala39Thr)	1
BARD1	2:215645982(G>T)	NM_000465.2(BARD1):c.616C>A (p.Gln206Lys)	1
BARD1	2:215645882(A>T)	NM_000465.4(BARD1):c.716T>A (p.Leu239Gln)	3
BARD1	2:215645699(G>A)	NM_000465.4(BARD1):c.899C>T (p.Pro300Leu)	1
BARD1	2:215645582(C>T)	NM_000465.4(BARD1):c.1016G>A (p.Ser339Asn)	1
BARD1	2:215633963(G>A)	NM_000465.4(BARD1):c.1388C>T (p.Thr463Ile)	1
BARD1	2:215617247(G>A)	NM_000465.4(BARD1):c.1601C>T (p.Thr534Ile)	1
BARD1	2:215609838(T>G)	NM_000465.4(BARD1):c.1856A>C (p.Lys619Thr)	1
BARD1	2:215593482(C>T)	NM_000465.4(BARD1):c.2252G>A (p.Arg751Gln)	1
BRCA1	17:41234489(G>A)	NM_007294.4(BRCA1):c.4289C>T (p.Pro1430Leu)	1
BRCA1	17:41219715(GAAA>GAA)	NM_007294.4(BRCA1):c.4987-4del	1
BRCA1	17:41219719(T>C)	NM_007294.4(BRCA1):c.4987-7A>G	1
BRCA2	13:32912901(TAAGAA>TAA)	NM_000059.4(BRCA2):c.4412_4414del (p.Arg1471del)	1
BRCA2	13:32914848(A>G)	NM_000059.3(BRCA2):c.6356A>G (p.Asn2119Ser)	1
BRCA2	13:32929345(A>T)	NM_000059.3(BRCA2):c.7355A>T (p.Asn2452Ile)	1
BRCA2	13:32931889(A>G)	NM_000059.4(BRCA2):c.7628A>G (p.Tyr2543Cys)	1
BRCA2	13:32937593(A>T)	NM_000059.4(BRCA2):c.8254A>T (p.Ile2752Phe)	1
BRCA2	13:32953903(G>C)	NM_000059.3(BRCA2):c.8970G>C (p.Trp2990Cys)	1

© 2024 Rezoug Z et al. *JAMA Network Open.*

BRCA2	13:32972320(A>G)	NM_000059.4(BRCA2):c.9670A>G (p.Ile3224Val)	1
BRIP1	17:59926582(A>C)	NM_032043.3(BRIP1):c.415T>G (p.Ser139Ala)	1
BRIP1	17:59886091(A>G)	NM_032043.3(BRIP1):c.655T>C (p.Cys219Arg)	1
BRIP1	17:59878690(G>A)	NM_032043.2(BRIP1):c.1064C>T (p.Ala355Val)	1
BRIP1	17:59858218(A>G)	NM_032043.2(BRIP1):c.1777T>C (p.Cys593Arg)	1
BRIP1	17:59820469(G>A)	NM_032043.3(BRIP1):c.2284C>T (p.Arg762Cys)	1
BRIP1	17:59820429(T>C)	NM_032043.3(BRIP1):c.2324A>G (p.Asn775Ser)	1
BRIP1	17:59820414(A>T)	NM_032043.3(BRIP1):c.2339T>A (p.Ile780Lys)	1
CDH1	16:68835772(C>A)	NM_004360.5(CDH1):c.363C>A (p.His121Gln)	1
CDH1	16:68842666(C>A)	NM_004360.5(CDH1):c.602C>A (p.Pro201His)	1
CDH1	16:68845655(G>A)	NM_004360.5(CDH1):c.901G>A (p.Ala301Thr)	1
CDH1	16:68853209(A>G)	NM_004360.5(CDH1):c.1592A>G (p.Asn531Ser)	1
CDH1	16:68855975(C>T)	NM_004360.5(CDH1):c.1783C>T (p.Pro595Ser)	1
CHEK2	22:29121339(G>C)	NM_007194.3(CHEK2):c.336C>G (p.Asn112Lys)	1
CHEK2	22:29121253(T>G)	NM_007194.4(CHEK2):c.422A>C (p.Lys141Thr)	1
CHEK2	22:29121112(C>T)	NM_007194.4(CHEK2):c.445G>A (p.Glu149Lys)	1
CHEK2	22:29121087(A>G)	NM_007194.4(CHEK2):c.470T>C (p.Ile157Thr)	4
CHEK2	22:29121019(G>A)	NM_007194.4(CHEK2):c.538C>T (p.Arg180Cys)	1
CHEK2	22:29120961(T>C)	NM_007194.4(CHEK2):c.592+4A>G	1
CHEK2	22:29092948(G>A)	NM_007194.4(CHEK2):c.1036C>T (p.Arg346Cys)	1
CHEK2	22:29091207(G>A)	NM_007194.4(CHEK2):c.1283C>T (p.Ser428Phe)	1
CHEK2	22:29090054(G>A)	NM_007194.4(CHEK2):c.1427C>T (p.Thr476Met)	2
CHEK2	22:29083920(TGG>TG)	NM_007194.4(CHEK2):c.1596del (p.Thr533fs)	1
MLH1	3:37059005(G>A)	NM_000249.4(MLH1):c.799G>A (p.Val267Ile)	1
MLH1	3:37067152(C>T)	NM_000249.4(MLH1):c.1063C>T (p.Pro355Ser)	1
MLH1	3:37067207(G>A)	NM_000249.4(MLH1):c.1118G>A (p.Gly373Glu)	1
MLH1	3:37067433(G>T)	NM_000249.4(MLH1):c.1344G>T (p.Glu448Asp)	1
MLH1	3:37090429(G>C)	NM_000249.4(MLH1):c.2024G>C (p.Ser675Thr)	1
MSH2	2:47637418(C>A)	NM_000251.3(MSH2):c.552C>A (p.Phe184Leu)	1
MSH2	2:47672737(C>A)	NM_000251.3(MSH2):c.1327C>A (p.Leu443Ile)	1
MSH2	2:47709921(G>A)	NM_000251.2(MSH2):c.2638G>A(p.Gly880Ser)	1
MSH6	2:48010555(GCGC>GTGC)	NM_000179.3(MSH6):c.184C>T (p.Arg62Cys)	1
MSH6	2:48026281(G>A)	NM_000179.3(MSH6):c.1159G>A (p.Asp387Asn)	1
MSH6	2:48026842(T>A)	NM_000179.3(MSH6):c.1720T>A (p.Ser574Thr)	1
MSH6	2:48028030(T>A)	NM_000179.3(MSH6):c.2908T>C (p.Trp970Arg)	1
MSH6	2:48032740(ATTTTTTTTTTTTT>ATTTTTTTTTT)	NM_000179.2(MSH6):c.3557-6_3557-4delTTT	1
MSH6	2:48033763(A>G)	NM_000179.3(MSH6):c.3974A>G (p.Lys1325Arg)	1
PALB2	16:23647464(G>A)	NM_024675.3(PALB2):c.403C>T (p.Pro135Ser)	1
PALB2	16:23646330(T>C)	NM_024675.4(PALB2):c.1537A>G (p.Thr513Ala)	1
PALB2	16:23641584(AGCAGGACTT>A)	NM_024675.4(PALB2):c.1882_1890del (p.Lys628_Cys630del)	1
PALB2	16:23641026(T>C)	NM_024675.4(PALB2):c.2449A>G (p.Thr817Ala)	1
PALB2	16:23637690(A>G)	NM_024675.4(PALB2):c.2615T>C (p.Val872Ala)	1
PALB2	16:23619310(A>T)	NM_024675.4(PALB2):c.3225T>A (p.Ser1075Arg)	2
PALB2	16:23619288(C>T)	NM_024675.4(PALB2):c.3247G>A (p.Glu1083Lys)	1

© 2024 Rezoug Z et al. *JAMA Network Open.*

PTEN	10:89624312(A>G)	NM_000314.8(PTEN):c.79+7A>G	1
PTEN	10:89725230(ATTTTTTTTT>ATTTTTTTT)	NM_000314.8(PTEN):c.*10del	1
RAD51C	17:56770007(G>T)	NM_058216.3(RAD51C):c.3G>A (p.Met1Ile)	1
RAD51C	17:56798168(C>T)	NM_058216.3(RAD51C):c.899C>T (p.Ala300Val)	1
RAD51C	17:56811483(A>G)	NM_058216.3(RAD51C):c.1031A>G (p.Gln344Arg)	1
RAD51D	17:33434075(T>G)	NM_002878.4(RAD51D):c.412A>C (p.Asn138His)	1
RAD51D	17:33434054(G>A)	NM_002878.4(RAD51D):c.433C>T (p.Arg145Cys)	1
RAD51D	17:33430511(G>A)	NM_002878.4(RAD51D):c.629C>T (p.Ala210Val)	1
RAD51D	17:33430277(A>G)	NM_002878.4(RAD51D):c.734T>C (p.Val245Ala)	1
RAD51D	17:33428351(C>T)	NM_002878.4(RAD51D):c.772G>A (p.Gly258Arg)	1
RAD51D	17:33428327(G>A)	NM_002878.4(RAD51D):c.796C>T (p.Arg266Cys)	1
RAD51D	17:33428300(G>A)	NM_002878.4(RAD51D):c.823C>T (p.Arg275Trp)	1
RAD51D	17:33428290(A>G)	NM_002878.4(RAD51D):c.833T>C (p.Leu278Pro)	1
RAD51D	17:33428279(C>T)	NM_002878.4(RAD51D):c.844G>A (p.Glu282Lys)	1

ATM (NM_000051.4), BARD1 (NM_000465.4), BRCA1 (NM_007294.4), BRCA2 (NM_000059.4), BRIP1 (NM_032043.3), CDH1 (NM_004360.5), CHEK2 (NM_007194.4), EPCAM (NM_002354.3), MLH1 (NM_000249.4), MSH2 (NM_000251.3), MSH6 (NM_000179.3), PALB2 (NM_024675.4), PTEN (NM_000314.8), RAD51C (NM_058216.3) and RAD51D (NM_002878.4).

© 2024 Rezoug Z et al. *JAMA Network Open.*

**eTable 5.** Distribution of germline pathogenic/likely pathogenic variants and variants of uncertain significance by parental origin[†]			
**Region or Ethnicity**	**Population (% with Reported Variants)**	**Pathogenic (% of Population)**	**VUS (% of Population)**
Western Europe	208 (15.3%)	13 (6.2%)	19 (9.1%)
Eastern Europe	91 (17.6%)	5 (5.5%)	11 (12.1%)
Middle East	55 (38.2%)	4 (7.3%)	17 (30.9%)
North Africa	32 (12.5%)	2 (6.2%)	2 (6.2%)
East Asia	31 (35.5%)	2 (6.4%)	9 (29%)
South East Asia	29 (24.1%)	4 (13.8%)	3 (10.4%)
South Europe	26 (15.4%)	2 (7.7%)	2 (7.7%)
South and Central America	26 (19.2%)	3 (11.5%)	2 (7.7%)
South Asia	22 (31.8%)	3 (13.6%)	4 (18.2%)
Sub Saharan Africa	21 (23.8%)	0 (0%)	5 (23.8%)
Caribbean	20 (35%)	3 (15%)	4 (20%)
Central Asia	1 (100%)	0 (0%)	1 (100%)
Australia and New Zealand	1 (0.1%)	0 (0%)	0 (0%)
French Canadian	171 (21.6%)	15 (8.8%)	22 (12.9%)
Jewish	58 (6.9%)	1 (1.7%)	3 (5.2%)
Indigenous	8 (12.5%)	0 (0%)	1 (12.5%)

[†]If a patient reported ancestry from multiple regions/ethnicities they were counted in all those regions/ethnicities;

© 2024 Rezoug Z et al. *JAMA Network Open.*

eTable 6. Predictors of pathogenic or likely pathogenic variants in secondary 14-gene panel among women with newly diagnosed invasive breast cancer (n=659)

**Characteristic**	**Cohort (n=659)**	**Proportion with P/LP Variant on Secondary Panel**		**Unadjusted OR for P/LP on Secondary Panel**	
	**No. (%)**	**%**	***P value***	**OR**	**95% CI**
**Age Group**			0.33		
< 40 years	79 (12.0)	2.5		1.07	(0.20 – 5.60)
40-49 years	178 (27.0)	2.3		0.94	(0.25 – 3.56)
50-59 years	210 (31.8)	2.4		*Ref*	
60-69 years	138 (20.9)	5.8		2.52	(0.81 – 7.88)
70+ years	54 (8.2)	1.9		0.77	(0.09 – 6.76)
**Race/Ethnicity**			0.32		
White/European	432 (65.6)	2.4		-	
Black/African/Caribbean	29 (4.4)	0			
Asian/Southeast Asian	66 (10.0)	1.5			
Hispanic/South or Central American	22 (3.3)	0			
Middle Eastern/North African	63 (9.6)	0			
Indigenous/First Nations	2 (0.3)	0			
Other/Unknown	45 (6.8)	7.0			
**Ancestry**			0.63		
Ashkenazi Jewish	47 (7.1)	4.4		1.69	(0.37 - 7.81)
French Canadian	153 (23.2)	3.9		1.51	(0.56 - 4.10)
Non-Ashkenazi Jewish/French Canadian	459 (69.6)	2.6		*Ref*	
**Cancer-specific family history**			0.30		
No known family history of cancer	144 (21.8)	1.4		*Ref*	
Ovarian cancer (+/- breast cancer)	44 (6.7)	7.0		5.29	(0.85 - 32.7)
Breast cancer	269 (40.8)	3.0		2.15	(0.45 - 10.3)
Any non-breast/ovarian cancer	202 (30.7)	3.5		2.52	(0.52 - 12.)
**Laterality**			0.14		
Unilateral	634 (96.2)	2.8		*Ref*	
Synchronous bilateral	25 (3.8)	8.0		2.98	(0.65 - 13.6)
**Histology**			0.91		
Invasive ductal carcinoma	526 (79.8)	2.9		*Ref*	
Invasive lobular carcinoma	67 (10.2)	4.5		1.59	(0.45 - 5.66)
Mixed ductal and lobular	30 (4.6)	3.2		1.13	(0.15 - 8.87)
Other/Unknown	36 (5.4)	2.8		0.97	(0.13 - 7.57)
**Histologic Grade**			0.53		
Grade I	107 (16.2)	4.7		*Ref*	
Grade II	349 (53.0)	2.9		0.59	(0.20 - 1.78)
Grade III	203 (30.8)	2.5		0.51	(0.14 - 1.80)
**Biologic Subtype**			0.44		
ER+HER2-	442 (67.1)	3.4		*Ref*	
HER2+	118 (17.9)	3.4		1.00	(0.33 - 3.07)
TNBC	99 (15.0)	1.0		0.29	(0.04 - 2.23)
**Clinical Tumor Size***			0.68		
T1	363 (55.6)	3.3		*Ref*	
T2	233 (35.8)	2.1		0.64	(0.22 - 1.84)
T3-T4	56 (8.6)	3.6		1.08	(0.24 - 4.97)
**Clinical Nodal Status***			0.71		
N0	490 (74.4)	3.3		*Ref*	
N1	143 (22.8)	2.1		0.64	(0.18 - 2.22)
N2-N3	22 (3.4)	4.6		1.41	(0.18 - 11.2)
**Stage***			0.43		
Stage I	322 (48.9)	3.7		*Ref*	
Stage II	262 (39.8)	1.9		0.50	(0.17 - 1.44)
Stage III	45 (6.8)	4.4		1.20	(0.26 - 5.55)
Stage IV	23 (3.5)	0		-	
**Meet traditional testing criteria**			0.15		
No	464 (70.4)	3.7		*Ref*	
Yes	195 (29.6)	1.5		0.41	(0.12-1.42)

P/LP: Pathogenic/Likely pathogenic; ***TNBC:*** Triple negative breast cancer; *Patients with unknown data excluded from Chi-squared analysis.

© 2024 Rezoug Z et al. *JAMA Network Open.*

eTable 7. PARP inhibitor eligibility

Eligibility Criteria*	**Cohort** **(n=729)** No. candidates pre-genetic testing *(% total cohort)*	**PARPi-eligible** **(n=13)** No. candidates with a GPV in *BRCA1/2 (% biologic subtype who are eligible)*
**Triple Negative Breast Cancer (n=112)**	**64 (8.8)**	**12 (18.8)**
Neoadjuvant chemotherapy with residual disease	54 (7.4)	9 (14.1)
Primary surgery, at least pT2N0 or pN1	4 (0.6)	1 (1.6)
De novo metastatic	6 (0.8)	2 (3.1)
**ER+HER2- Breast Cancer (n=487)**	**37 (5.1)**	**1 (2.7)**
Neoadjuvant chemotherapy with CPS+EG score >/=3	8 (1.1)	0
Primary surgery, pN2+	16 (2.2)	0
De novo metastatic	13 (1.8)	1 (2.7)

GPV Germline pathogenic variant; ***PARPi*** Poly(ADP-ribose)polymerase inhibitor; ***TNBC*** Triple negative breast cancer; *Eligibility criteria include all patients with early-stage disease meeting OlympiA eligibility criteria (TNBC with residual disease after neoadjuvant chemotherapy or primary surgery with at least pT2 or pN1 disease; ER+HER2- breast cancer after neoadjuvant chemotherapy with a CPS+EG score of ≥3 or primary surgery with at least pN2 disease), as well as all patients with metastatic HER2-negative breast cancer.

© 2024 Rezoug Z et al. *JAMA Network Open.*

eTable 8. Clinical characteristics of patients stratified by phase I and phase II of the study (n=729)

Characteristic	Phase I (n=525)	Phase II (n=204)	p-value
**Age Group – *n, (%)***			0.03
<40 years	63 (12.0)	25 (12.3)	
40-49 years	136 (25.9)	60 (29.4)	
50-59 years	158 (30.1)	70 (34.3)	
60-69 years	112 (21.3)	42 (20.6)	
70+ years	56 (10.7)	7 (3.4)	
**Race/Ethnicity – *n,* (%)**			0.46
White/European	345 (65.7)	132 (64.7)	
Black/African/Caribbean	23 (4.4)	9 (4.4)	
Asian/Southeast Asian	53 (10.1)	23 (11.3)	
Hispanic/South or Central American	16 (3.1)	6 (2.9)	
Middle Eastern/North African	45 (8.6)	25 (12.3)	
Indigenous/First Nations	3 (0.6)	0 (0)	
Other/unknown	40 (7.6)	9 (4.4)	
**Ancestry – *n*, (%)**			0.13
Ashkenazi Jewish	36 (6.9)	18 (8.8)	
French Canadian	112 (21.3)	55 (27.0)	
Other	377 (71.8)	131 (64.2)	
**Family History – *n*, (%)**			0.29
No known family history of cancer	125 (23.8)	39 (19.1)	
Ovarian cancer (+/- breast cancer)	36 (6.9)	13 (6.4)	
Breast cancer	203 (38.7)	94 (46.1)	
Any non-breast/ovarian cancer	161 (30.7)	58 (28.4)	
**Laterality – *n,* (%)**			0.69
Unilateral	507 (96.6)	195 (95.6)	
Synchronous bilateral	18 (3.4)	9 (4.4)	
**Histology – *n,* (%)**			0.27
Invasive ductal carcinoma	412 (78.5)	172 (84.3)	
Invasive lobular carcinoma	58 (11.1)	15 (7.4)	
Mixed invasive ductal/lobular carcinoma	23 (4.4)	9 (4.4)	
Other/unknown histology	32 (6.1)	8 (3.9)	
**Grade – *n*, (%)**			0.17
Grade I	90 (17.1)	24 (11.8)	
Grade II	276 (52.6)	110 (53.9)	
Grade III	159 (30.3)	70 (34.3)	
**Biologic Subtype – *n*, (%)**			0.05
ER+HER2-	359 (68.4)	128 (62.8)	
HER2+	96 (18.3)	34 (16.7)	
TNBC	70 (13.3)	42 (20.6)	
**Clinical Tumor Size – *n*, (%)**			0.58
cT1	279 (53.1)	110 (53.9)	
cT2	195 (37.1)	69 (33.8)	
cT3-T4	44 (8.4)	23 (11.3)	
Unknown	7 (1.3)	2 (1.0)	
**Clinical Nodal Status – *n,* (%)**			0.91
cN0	391 (74.5)	150 (73.5)	
cN1	113 (21.5)	46 (22.6)	
cN2-N3	16 (3.1)	7 (3.4)	
Unknown	5 (1.0)	1 (0.5)	
**Anatomic Stage at Presentation – *n*, (%)**			0.63
Stage I	251 (47.8)	94 (46.1)	
Stage II	216 (41.1)	82 (40.2)	
Stage III	31 (5.9)	18 (8.8)	
Stage IV	22 (4.2)	7 (3.4)	
Unknown	5 (1.0)	3 (1.5)	

© 2024 Rezoug Z et al. *JAMA Network Open.*

eTable 9. Biologic subtype by age group (n=729)

	**<40 years (n=88)**	**40-49 years (n=196)**	**50-59 years (n=228)**	**60-69 years (n=154)**	**70+ years (n=63)**
**Biologic Subtype – *n, (%)***					
ER+HER2-	40 (45.5)	140 (71.4)	160 (70.2)	102 (66.2)	45 (71.4)
HER2+	24 (27.3)	39 (19.9)	39 (17.1)	24 (15.6)	4 (6.4)
TNBC	24 (27.3)	17 (8.7)	29 (12.7)	28 (18.2)	14 (22.2)

© 2024 Rezoug Z et al. *JAMA Network Open.*

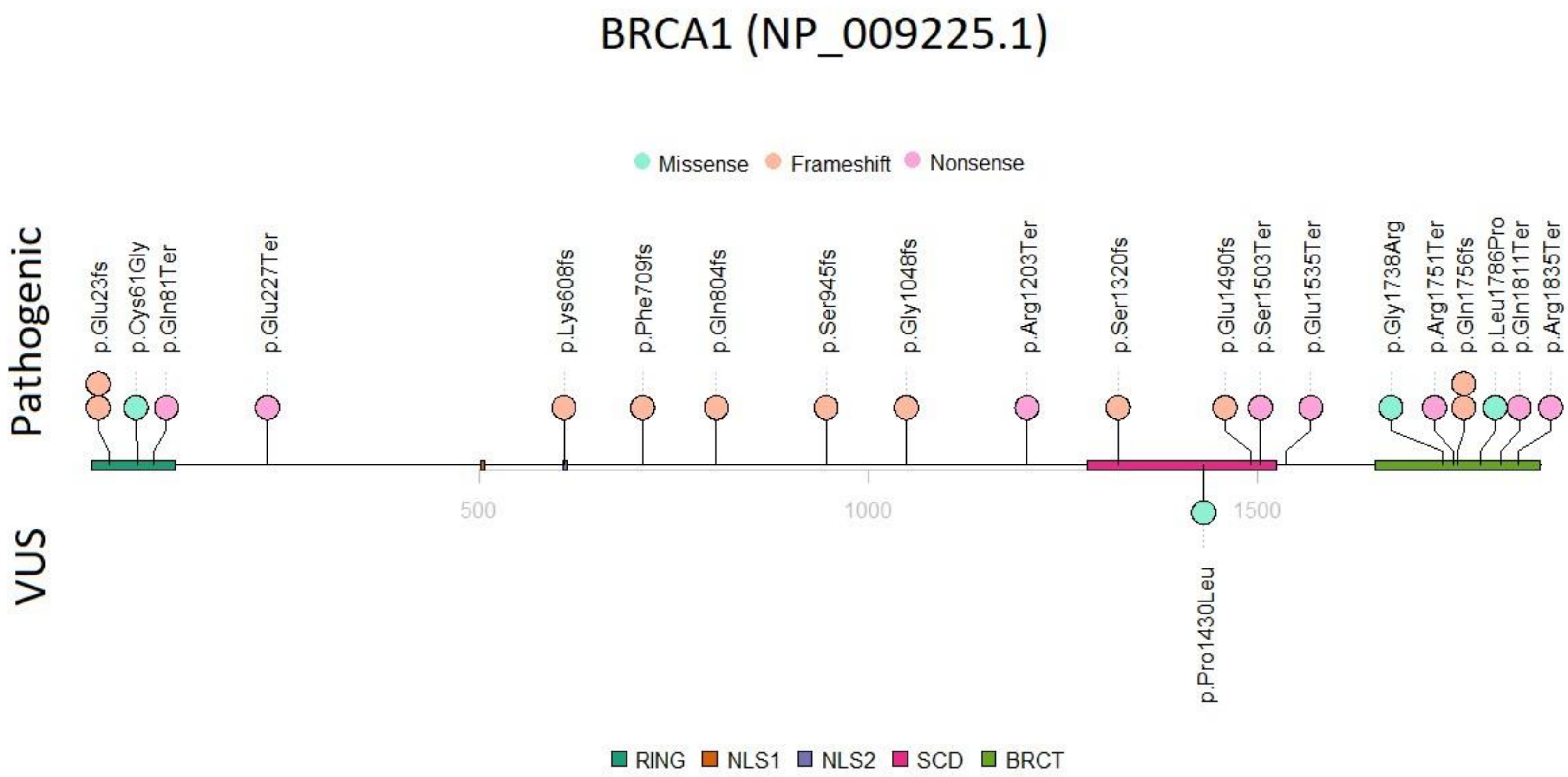

eFigure 1A. Lollipop plot for *BRCA1* germline variants. **Top** – GPVs, **Bottom** - VUSs. Domains: **RING** – RING finger containing domain, **NLS** 1 & 2 - nuclear localization sequences, **SCD** - Serine cluster domain, **BRCT** - BRCA1 C-terminal domain.

© 2024 Rezoug Z et al. *JAMA Network Open.*

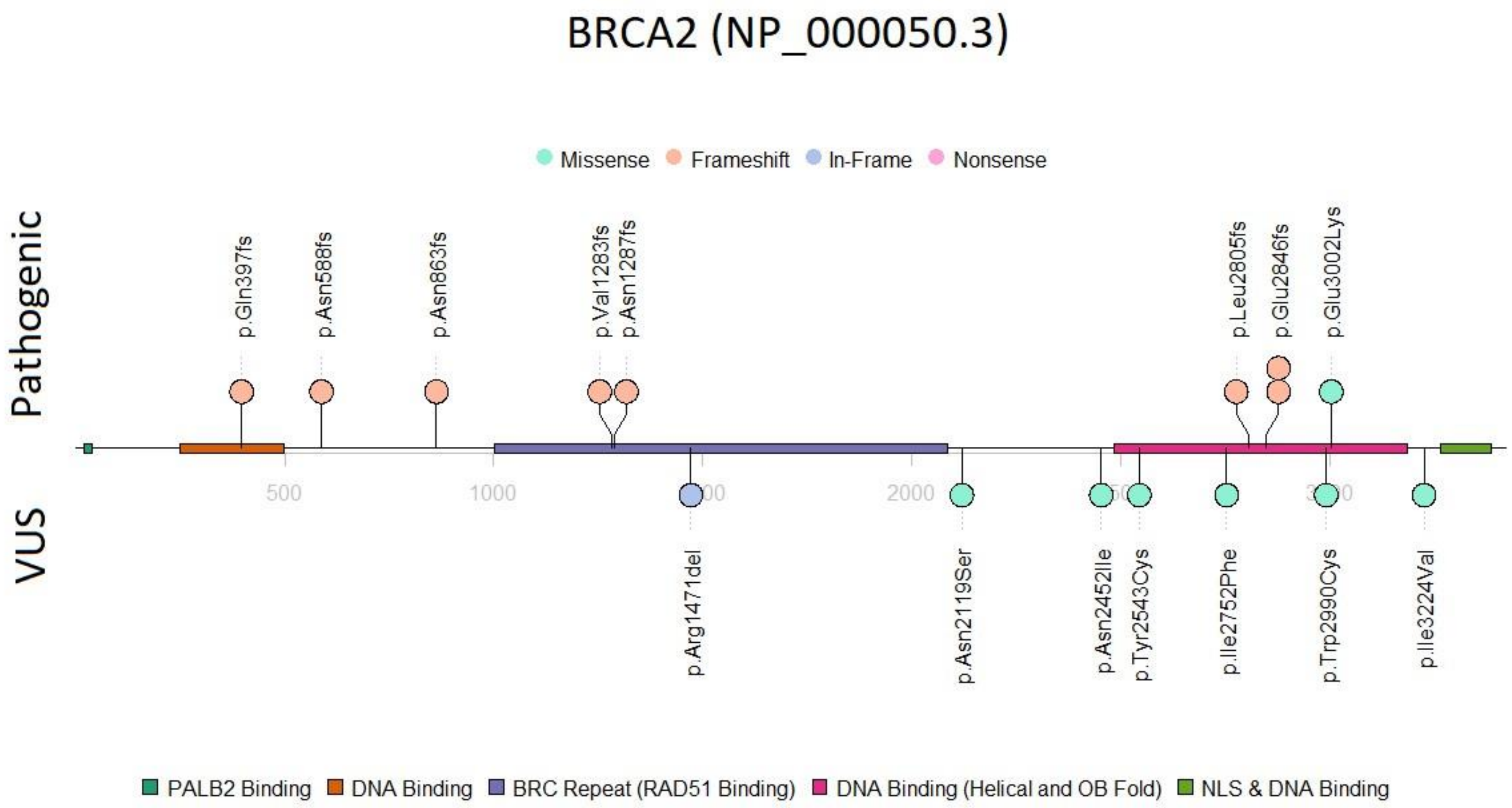

eFigure 1B. Lollipop plot for *BRCA2* germline variants. **Top** – GPVs, **Bottom** - VUSs.

© 2024 Rezoug Z et al. *JAMA Network Open.*

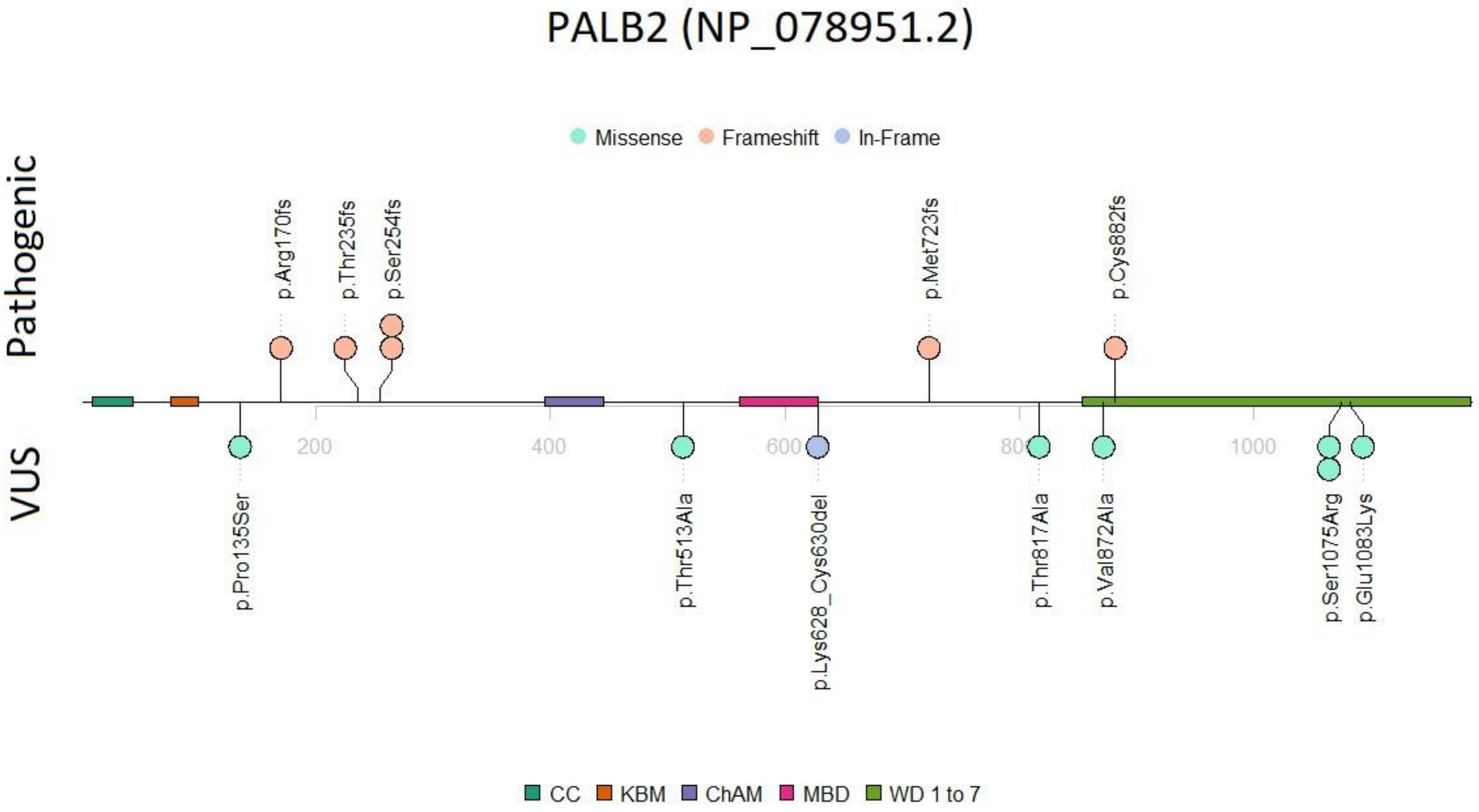

eFigure 1C. Lollipop plot for *PALB2* germline variants. **Top** – GPVs, **Bottom** - VUSs. Domains: CC- coiled-coil, KBM -KEAP1-binding motif, ChAM – Chromatin-association motif, **WD** 1 to 7 – beta-transducin repeats 1 to 7.

© 2024 Rezoug Z et al. *JAMA Network Open.*

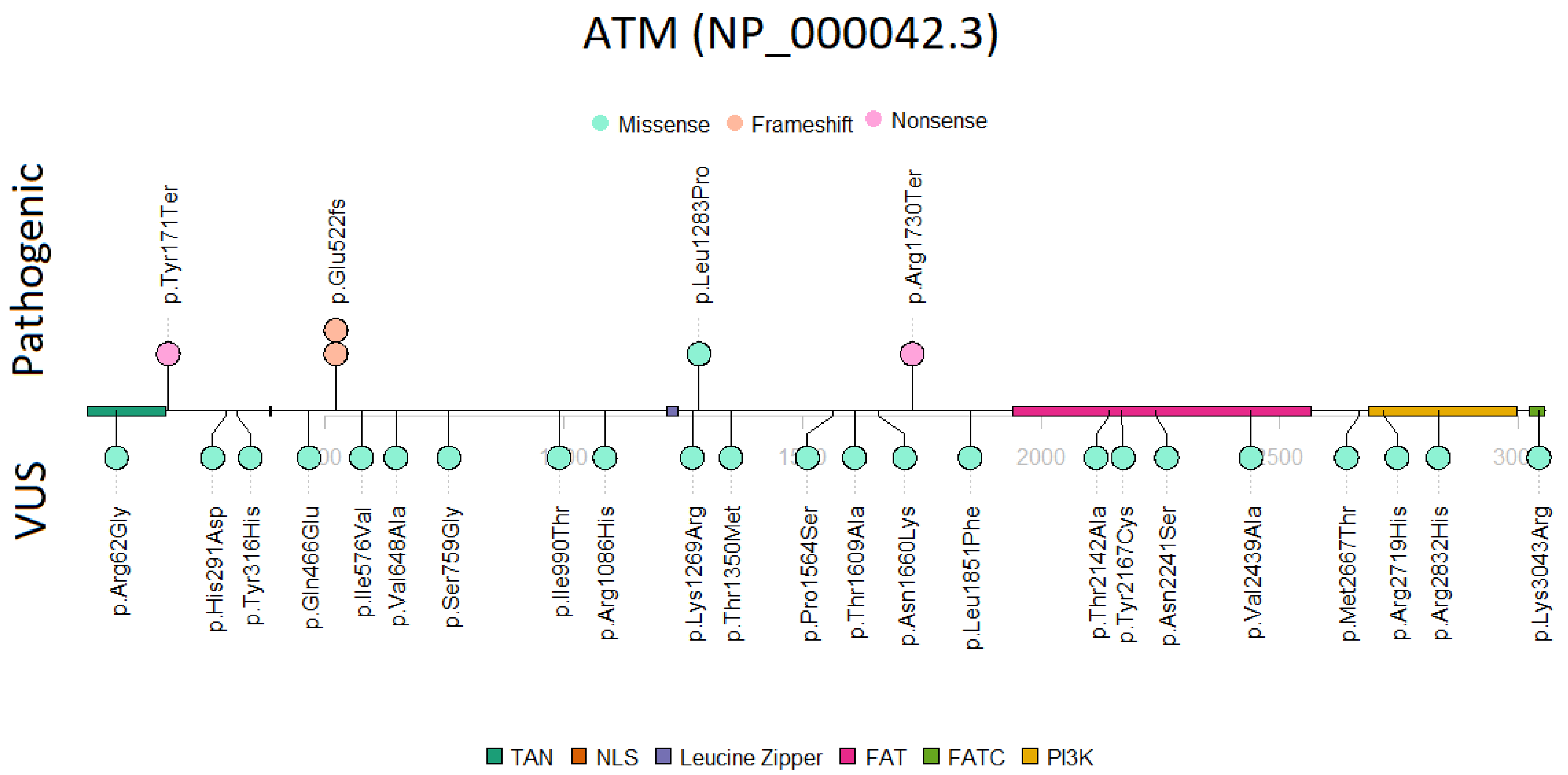

eFigure 1D. Lollipop plot for *ATM* germline variants. **Top** – GPVs, **Bottom** - VUSs. Domains: **TAN** - Tel1/ATM N-terminal motif, **NLS** - nuclear localization signal, **FAT** – FRAP ATM TRRAP domain, **FATC** – FAT C terminal domain, **PI3K** - PI3K/PI4K catalytic domain.

© 2024 Rezoug Z et al. *JAMA Network Open.*

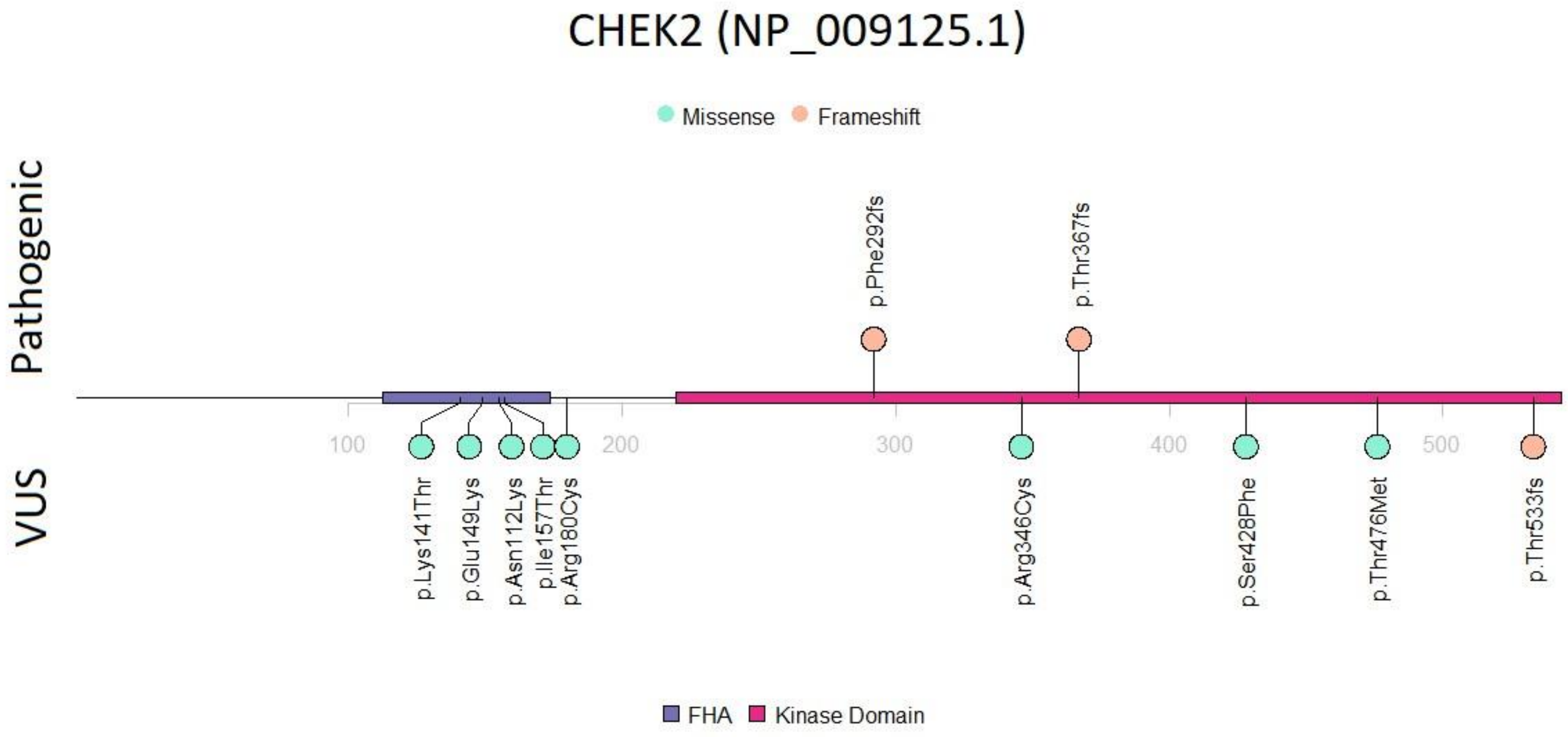

eFigure 1E. Lollipop plot for *CHEK2* germline variants. **Top** – GPVs, **Bottom** - VUSs. Domains: **FHA** - Central fork head associated domain.

© 2024 Rezoug Z et al. *JAMA Network Open.*

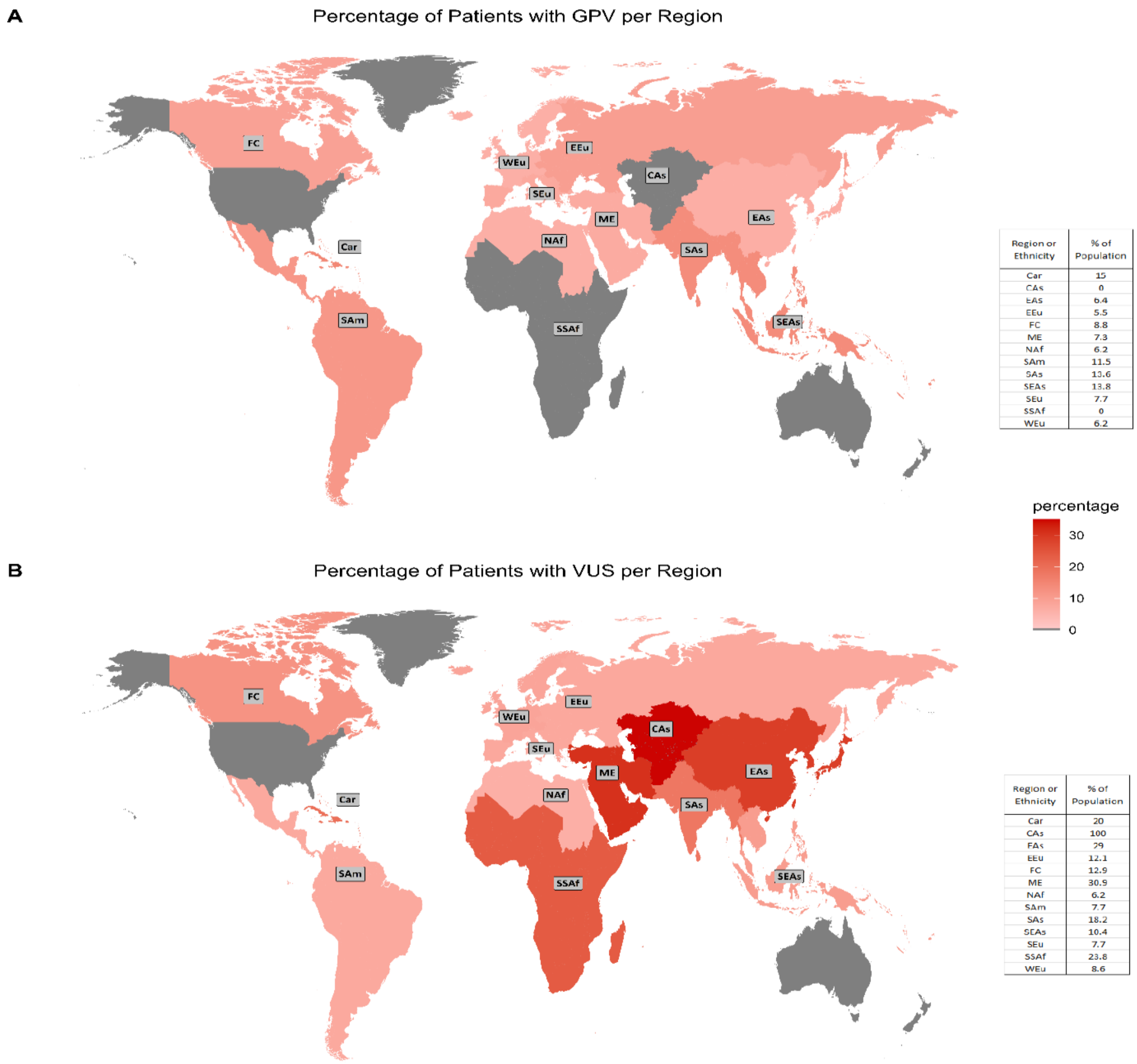

eFigure 2. World map showing ethnicity of patients with a pathogenic variant or variant of uncertain significance, per region. A) Number of pathogenic variants divided by number of patients per region; B) Number of variants of uncertain significance divided by number of patients, per region. **Car** – Caribbean, **CAs** – Central Asia, **EAs** – East Asia, **EEu** – Eastern Europe, **FC** - French Canadian, **ME** – Middle East, **NAf** – North Africa, **Sam** – South and Central America, **SAs** – South Asia, **SEAs** – South East Asia, **SEu** – South Eastern Europe, **SSAf** – Sub Saharan Africa, **WEu** – Western Europe.

© 2024 Rezoug Z et al. *JAMA Network Open.*

eReferences.

1. Abeliovich, D., et al., *The founder mutations 185delAG and 5382insC in BRCA1 and 6174delT in BRCA2 appear in 60% of ovarian cancer and 30% of early-onset breast cancer patients among Ashkenazi women.* Am J Hum Genet, 1997. **60**(3): p. 505-14.
2. Nguyen-Dumont, T., et al., *Genetic testing in Poland and Ukraine: should comprehensive germline testing of BRCA1 and BRCA2 be recommended for women with breast and ovarian cancer?* Genet Res (Camb), 2020. **102**: p. e6.
3. Oros, K.K., et al., *Significant proportion of breast and/or ovarian cancer families of French Canadian descent harbor 1 of 5 BRCA1 and BRCA2 mutations.* International Journal of Cancer, 2004. **112**(3): p. 411-419.
4. Belanger, M.H., et al., *A targeted analysis identifies a high frequency of BRCA1 and BRCA2 mutation carriers in women with ovarian cancer from a founder population.* J Ovarian Res, 2015. **8**: p. 1.
5. Tonin, P.N., et al., *Founder BRCA1 and BRCA2 mutations in French Canadian breast and ovarian cancer families.* Am J Hum Genet, 1998. **63**(5): p. 1341-51.
6. Konstantopoulou, I., et al., *High prevalence of BRCA1 founder mutations in Greek breast/ovarian families.* Clinical Genetics, 2014. **85**(1): p. 36-42.
7. Rashid, M.U., et al., *Prevalence of BRCA1 and BRCA2 mutations in Pakistani breast and ovarian cancer patients.* Int J Cancer, 2006. **119**(12): p. 2832-9.
8. Gomes, R., et al., *Haplotypic characterization of BRCA1 c.5266dupC, the prevailing mutation in Brazilian hereditary breast/ovarian cancer.* Genet Mol Biol, 2020. **43**(2): p. e20190072.
9. Krajc, M., et al., *Five recurrent BRCA1/2 mutations are responsible for cancer predisposition in the majority of Slovenian breast cancer families.* BMC Med Genet, 2008. **9**: p. 83.
10. Cote, S., et al., *The BRCA2 c.9004G>A (E2002K) [corrected] variant is likely pathogenic and recurs in breast and/or ovarian cancer families of French Canadian descent.* Breast Cancer Res Treat, 2012. **131**(1): p. 333-40.
11. Dansonka-Mieszkowska, A., et al., *A novel germline PALB2 deletion in Polish breast and ovarian cancer patients.* BMC Med Genet, 2010. **11**: p. 20.
12. Leedom, T.P., et al., *Breast cancer risk is similar for CHEK2 founder and non-founder mutation carriers.* Cancer Genetics, 2016. **209**(9): p. 403-407.
13. Ghadirian, P., et al., *The contribution of founder mutations to early-onset breast cancer in French-Canadian women.* Clinical Genetics, 2009. **76**(5): p. 421-426.
14. Shaag, A., et al., *Functional and genomic approaches reveal an ancient CHEK2 allele associated with breast cancer in the Ashkenazi Jewish population.* Hum Mol Genet, 2005. **14**(4): p. 555-63.
15. Cybulski, C., et al., *A novel founder CHEK2 mutation is associated with increased prostate cancer risk.* Cancer Res, 2004. **64**(8): p. 2677-9.
16. Laitman, Y., et al., *Germline CHEK2 mutations in Jewish Ashkenazi women at high risk for breast cancer.* Isr Med Assoc J, 2007. **9**(11): p. 791-6.
17. Rivera, B., et al., *Functionally Null RAD51D Missense Mutation Associates Strongly with Ovarian Carcinoma.* Cancer Research, 2017. **77**(16): p. 4517-4529.
18. Noskowicz, M., et al., *Prevalence of PALB2 mutation c.509_510delGA in unselected breast cancer patients from Central and Eastern Europe.* Fam Cancer, 2014. **13**(2): p. 137-42.
19. Oliver AW, Swift S, Lord CJ, Ashworth A, Pearl LH. *Structural basis for recruitment of BRCA2 by PALB2. EMBO Rep*. Sep 2009;10(9):990-6. doi:10.1038/embor.2009.126

© 2024 Rezoug Z et al. *JAMA Newtwork Open.*
