# Supplementary material for: Universal Genetic Testing for Newly Diagnosed Invasive Breast Cancer
Source: JAMA Netw Open. 2024 Sep 3;7(9):e2431427. doi: 10.1001/jamanetworkopen.2024.31427 (PMC11372499; doi:10.1001/jamanetworkopen.2024.31427)
Supplement: Supplement 2. — Data Sharing Statement [file jamanetwopen-e2431427-s002.pdf]

Data Sharing Statement

Rezoug. Universal Genetic Testing for Newly Diagnosed Invasive Breast Cancer. *JAMA Netw Open*. Published September 03, 2024. doi:10.1001/jamanetworkopen.2024.31427

Data
Data available: No

Additional Information
Explanation for why data not available: We will not provide germline pathogenic variant results linked to each patient, but we do provide a list of all germline pathogenic variants and VUSs found. We will provide a data dictionary on request. We will provide summary data beyond what is provided on request.
